# Spatial Genetic Structure and Demographic History of the Dominant Forest Oak *Quercus fabri* Hance in Subtropical China

**DOI:** 10.3389/fpls.2020.583284

**Published:** 2021-02-04

**Authors:** Xiao-Dan Chen, Jia Yang, Yu-Fan Guo, Yue-Mei Zhao, Tao Zhou, Xiao Zhang, Miao-Miao Ju, Zhong-Hu Li, Gui-Fang Zhao

**Affiliations:** ^1^Key Laboratory of Resource Biology and Biotechnology in Western China (Ministry of Education), College of Life Sciences, Northwest University, Xi’an, China; ^2^School of Biological Sciences, Guizhou Education University, Guiyang, China; ^3^School of Pharmacy, Xi’an Jiaotong University, Xi’an, China

**Keywords:** ecological differentiation, evolutionary history, intraspecific divergence, *Quercus*, subtropical China

## Abstract

Oak trees (*Quercus* L.) are important models for estimating abiotic impacts on the population structure and demography of long life span tree species. In this study, we generated genetic data for 17 nuclear microsatellite loci in 29 natural populations of *Quercus fabri* to estimate the population genetic structure. We also integrated approximate Bayesian computation (ABC) and ecological niche analysis to infer the population differentiation processes and demographic history of this oak species. The genetic analyses indicated two genetic clusters across the 29 populations collected, where most approximately corresponded to the intraspecific differentiation among populations from western and eastern China, whereas admixed populations were mainly found in central mountains of China. The best model obtained from hierarchical ABC simulations suggested that the initial intraspecific divergence of *Q. fabri* potentially occurred during the late Pliocene (*ca.* 3.99 Ma) to form the two genetic clusters, and the admixed population group might have been generated by genetic admixture of the two differentiated groups at *ca.* 53.76 ka. Ecological analyses demonstrated clear differentiation among the *Q. fabri* population structures, and association estimations also indicated significant correlations between geography and climate with the genetic variation in this oak species. Our results suggest abiotic influences, including past climatic changes and ecological factors, might have affected the genetic differentiation and demographic history of *Q. fabri* in subtropical China.

## Introduction

Historical and ecological factors have left complex imprints on the genetic structure and demographic history of extant species (e.g., Petit et al., 2002; Chen et al., 2008; Zhang et al., 2019). These factors include geographical and/or climatic processes, such as the uplift of the mountains, morphological reconstruction, and climatic oscillations associated with ice ages. The interactions among these processes over varying temporal scales may have led to the non-random distributions of plant species within or throughout landscapes (Eiserhardt et al., 2011). Indeed, spatial and temporal changes in these abiotic factors could have influenced genetic variation and population structures by creating physical barriers and novel ecological niches during the evolutionary history of species (e.g., Harmon et al., 2008; Ortego et al., 2012, 2015; Liu et al., 2013; Sun et al., 2015; Du et al., 2017; Liu et al., 2019a,b). Previous studies have suggested that local adaptation to varying climate or ecological niches was a major driver of the geographical patterns and current genetic structure of plant species (Ortego et al., 2015; Hipp et al., 2018). In addition, various ecological factors such as precipitation and temperature may have been responsible for driving intraspecific differentiation (Yang et al., 2018; Li et al., 2019).

In China, the subtropical zone covers the area from 22 to 34°N, and it is characterized as a heterogeneous environment with complex topography (Qiu et al., 2011). It is considered that this region was not influenced by the massive ice sheet during the glaciation period, but it was affected by episodic uplifts of the Qinghai–Tibet Plateau (QTP) during the late Pliocene to the early Pleistocene and associated glaciation events during the Quaternary (Shi et al., 1986; Fu et al., 2014). In fact, the rise of the QTP dramatically modified the global climate (Ruddiman and Kutzbach, 1989; Shi et al., 1999), including triggering and intensifying the Asian monsoon, furthermore, and this process also significantly affected the evolution of plants (Liu et al., 2013). In addition, the rise of QTP remodeled the geomorphology of China and changed the terrain into a hypsographic ladder in three steps ranging from high in the west to low in the east, thereby causing the climatic heterogeneity between western and eastern China and the longitudinal differentiation of the flora in this region (e.g., Sun et al., 2014; Zhang et al., 2018).

More recent climatic fluctuations during the Quaternary may also have affected intraspecific differentiation because populations could have contracted into refugia during glacial periods. These climate oscillations influenced the population demography by causing range shifts and possibly driving local adaptation (Ye et al., 2019). Phylogeographic analyses at intraspecific levels have suggested that the population genetic structure and demography were also profoundly affected by the complex geographical, climatic, and ecological factors in subtropical China (Shi et al., 2014; Sun et al., 2014; Gong et al., 2016; Ye et al., 2019). However, these previous studies focused on the evergreen broad-leaved forest (EBLF) because of its high abundance, and thus our understanding is unclear regarding how the interplay between topography and climatic events led to the intraspecific differentiation of deciduous tree species in this area and their evolutionary patterns.

*Quercus* species are regarded as powerful models for studying the adaptation of forest trees to variable environments because of their wide geographical range and the large variations in the climatic and edaphic conditions in the areas that they occupy (Gailing et al., 2009; Sork et al., 2010; Lagache et al., 2013; Cavender-Bares, 2019). The genus *Quercus* comprises more than 400 species, and the highest diversity is observed in the Americas (*ca.* 220 species), particularly in Mexico (*ca.* 161 species) and Southeast Asia (*ca.* 130–160 species) (Menitsky, 1984; Luo and Zhou, 2000; Valencia-Ávalos, 2004). Section *Quercus* accounts for most of the species in North America and about 30 species in Eurasia (Denk et al., 2017). *Quercus fabri* belongs to section *Quercus*, and it is an economically important and dominant deciduous tree species distributed in subtropical China. The hardwood obtained from *Q. fabri* is an excellent material for constructing furniture and flooring. This species is regarded as a pioneer species during secondary succession in the recovery of EBLF in China due to its ability to grow in barren soils, as well as its high capacity for sprouting and tolerance of environmental disturbance. In addition, *Q. fabri* is used to control soil erosion in mountainous areas of subtropical China (Wu et al., 2005; He et al., 2008; Shi et al., 2019). Moreover, *Q. fabri* is an important component of urban landscapes in China because of its colorful leaves. Thus, *Q. fabri* is an ecologically and economically important tree species, but its populations occur in patches of forests fragmented by agriculture and human disturbance, and thus, there is an urgent need to conserve this endemic oak species in China.

A previous phylogeographic study showed that *Q. fabri* might have contracted into refugia during the glacial period and expanded in the warm period (Chen et al., 2020). Evolutionary simulations based on several chloroplast makers also suggested that the intraspecific differentiation of this oak species may date back to the late Pliocene but with a weak phylogeographic structure (Chen et al., 2020). However, previous research into the evolutionary history of this species was limited by the sample size employed (293 individuals). Due to the widespread distribution and dominant position of subtropical deciduous forests in China. *Q. fabri* may be useful model for evaluating the impact of the stepped geomorphology in subtropical China on the spatial genetic structure and demographic history of deciduous tree species.

In the current study, we used 17 nuclear simple sequence repeat (SSR) loci to assess the genetic variation in *Q. fabri* based on 29 populations (490 individuals) sampled throughout its distribution range in subtropical China. The special goals of this study were to (1) determine the genetic diversity and population structure of *Q. fabri*, (2) infer its intraspecific evolutionary history and evaluate the effects of geological and/or past climatic factors, and (3) test whether environment factors (geography and climate) contributed to the current distribution.

## Materials and Methods

### Population Sample and DNA Extraction

**FIGURE 1:**
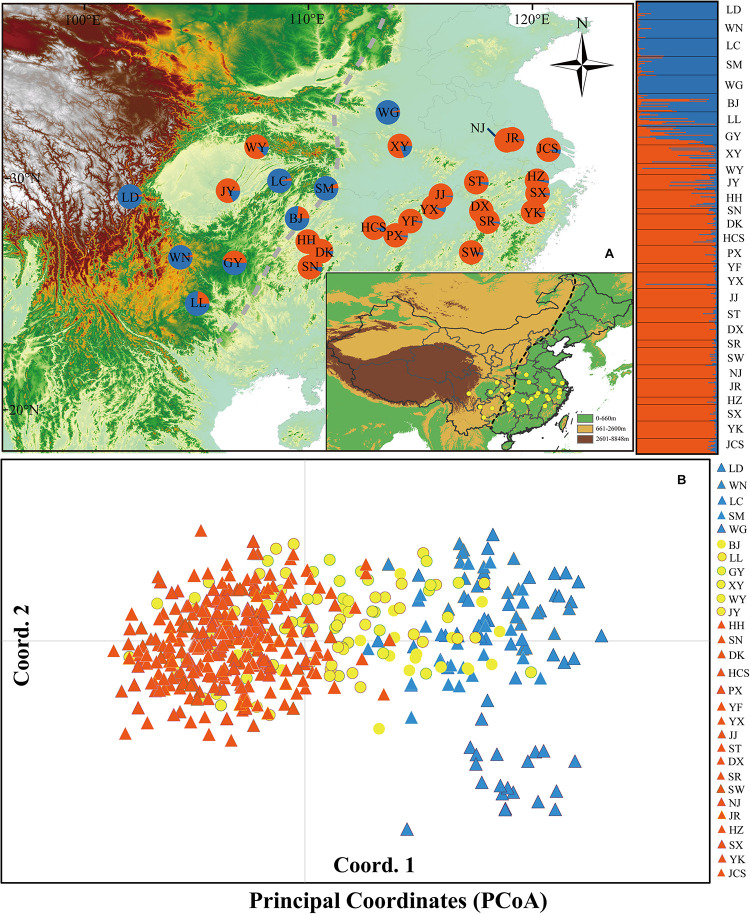
**(A)** Color-coded groupings of 29 *Quercus fabri* populations according to the structure with the most likely group number, *K* = 2. **(B)** Principal coordinates analysis (PCoA). The dashed line represents the boundary between the second steppe region and the third steppe region in China. The blue and orange triangles represent the populations of group 1 and group 3, respectively; the yellow circles represent the populations of group 2.

We sampled leaf material from 29 populations covering the range of *Q. fabri* in subtropical China. At each site, mature foliage was sampled from 6 to 21 individuals spaced > 100 m apart and dried rapidly with silica gel, before storing at −10°C until DNA isolation, where 490 individuals were sampled in total (Table 1 and Figure 1). Voucher specimens of each population were stored in the Evolutionary Botany Lab of Northwest University. Total genomic DNA was isolated using a Plant Genomic DNA Extraction Kit (Tiangen, Beijing, China).

**TABLE 1:** Genetic diversity of 29 populations of *Quercus fabri* estimated based on 17 microsatellite loci.

Pop	Location	Latitude	Longitude	*N*	*N*a	*N*e	*I*	*H*_O_	*H*_E_	*PPL* (%)	*F*_IS_	Qp
		(°N)	(°E)									
LD	Luding, Sichuan	29.57	102.02	19	4.824	2.972	1.083	0.383	0.533	94.12	0.306	0.95
WN	Weining, Guizhou	26.86	104.28	20	5.176	2.833	1.044	0.350	0.497	94.12	0.319	0.95
LC	Enshilichuan, Hubei	30.27	108.70	20	5.706	3.606	1.278	0.444	0.609	94.12	0.296	0.95
SM	Shimen, Hunan	29.94	110.78	20	6.176	3.878	1.312	0.370	0.601	94.12	0.406	0.80
WG	Wugang, Henan	33.33	113.54	20	4.941	3.123	1.108	0.341	0.548	88.24	0.400	0.95
BJ	Baojing, Hunan	28.60	109.49	20	6.412	3.771	1.317	0.400	0.604	94.12	0.361	0.70
LL	Longlin, Guangxi	24.79	105.03	16	5.529	3.959	1.288	0.419	0.609	94.12	0.341	0.56
GY	Guiyang, Guizhou	26.61	106.69	20	6.176	3.780	1.289	0.380	0.591	94.12	0.379	0.10
XY	Xinayang, Henan	31.83	114.08	20	5.294	3.390	1.189	0.367	0.570	100.00	0.378	0.60
WY	Wanyuan, Sichuan	31.80	107.68	12	4.176	2.527	0.954	0.329	0.482	88.24	0.356	0.75
JY	Jinyun, Sichuan	29.83	106.40	17	5.294	3.086	1.160	0.349	0.562	94.12	0.407	0.47
HH	Huaihua, Hunan	27.56	109.96	20	6.000	3.615	1.236	0.384	0.574	100.00	0.355	0.80
SN	Suining, Hunan	26.42	110.06	6	3.353	2.609	0.902	0.373	0.484	82.35	0.314	1
DK	Hongjiangdongkou,Hunan	27.13	110.56	17	5.529	3.285	1.139	0.397	0.525	94.12	0.273	0.88
HCS	Changsha, Hunan	28.18	112.93	17	6.118	3.617	1.207	0.360	0.538	94.12	0.357	0.88
PX	Pingxiang, Jiangxi	27.84	113.89	20	6.353	3.490	1.186	0.339	0.528	100.00	0.380	0.95
YF	Yifeng, Jiangxi	28.48	114.55	9	4.471	2.904	1.015	0.373	0.494	94.12	0.300	1
YX	Yongxiu, Jiangxi	29.10	115.59	18	5.706	3.313	1.161	0.344	0.538	94.12	0.386	0.89
JJ	Jiujiang, Jiangxi	29.61	115.91	21	6.235	3.519	1.191	0.454	0.541	100.00	0.185	1
ST	Shitai, Anhui	30.21	117.49	16	6.176	3.919	1.239	0.441	0.555	94.12	0.236	0.94
DX	Dexing, Jiangxi	28.93	117.72	19	5.059	3.251	1.058	0.380	0.499	94.12	0.261	1
SR	Shangrao, Jiangxi	28.45	117.98	8	4.765	3.147	1.100	0.390	0.532	88.24	0.328	1
SW	Shaowu, Fujian	27.08	117.27	19	6.118	3.702	1.208	0.410	0.547	94.12	0.277	1
NJ	Nanjiang, Jiangsu	32.08	118.85	15	5.294	3.398	1.172	0.319	0.559	100.00	0.458	0.93
JR	Jurong, Jiangsu	32.13	119.09	20	5.824	3.520	1.175	0.281	0.535	94.12	0.494	0.95
HZ	Hangzhou, Zhejiang	30.28	120.20	8	3.824	2.804	0.949	0.309	0.479	94.12	0.413	0.88
SX	Shaoxing, Zhejiang	29.71	120.23	18	5.706	3.501	1.137	0.383	0.518	94.12	0.287	1
YK	Yongkang, Zhejiang	28.88	120.03	19	5.294	3.105	1.069	0.312	0.495	94.12	0.393	1
JCS	Changshu, Jiangsu	31.66	120.72	16	5.353	3.372	1.203	0.365	0.575	94.12	0.393	0.81
Mean					5.410	3.345	1.151	0.371	0.542	94.12	0.346	

*N, sample size; Na, the number of alleles; Ne, number of effective alleles; I, Shannon’s Information Index; H_*O*_, observed heterozygosity; H_*E*_, expected heterozygosity; PPL, percentage of polymorphic loci; F_*IS*_, inbreeding coefficient; Qp, proportion of genetically pure individuals in each population.*

### PCR Amplification and Simple Sequence Repeat Genotyping

All 490 samples were amplified at 17 SSR nuclear loci (see details in Supplementary Note 1 and Supplementary Table 1). PCR amplification was performed in a total volume of 20 μl comprising 11 μl of 2 × Taq PCR MasterMix, 0.3 μl of each primer, 1 μl of primer template (10–50 ng), and 7.4 μl double-distilled H_2_O. All amplifications were conducted using a PTC-2000 thermal cycler (MJ Research) as follows: 5 min at 94°C, followed by 32 cycles at 94°C for 40 s, at the specific annealing temperature (Tm) for each marker (Supplementary Table 1), and at 72°C for 90 s, with a final extension at 72°C for 10 min. The final PCR products were sequenced using an ABI 3730 XL Analyzer (Applied Biosystems, Foster City, CA, United States) and analyzed with GENEMARKER v2.2.0 (Holland and Parson, 2011).

### Genetic Diversity Analysis

MICROCHECKER v2.2.3 (Van Oosterhout et al., 2004) was used to test the presence of null alleles for all loci. Linkage disequilibrium (LD) and departure from Hardy–Weinberg equilibrium were evaluated using POPGENE v1.32 (Yeh et al., 1999). Genetic diversity indices (genetic diversity within populations, *H*_S_; total genetic diversity, *H*_T_) were obtained for each microsatellite locus with FSTAT v2.9.3 (Goudet, 2001). We also calculated the observed heterozygosity (*H*_O_), expected heterozygosity (*H*_E_), and polymorphism information content for each microsatellite locus with CERVUS v3.0.7 (Marshall et al., 1998). GENALEX v6.5 (Peakall and Smouse, 2012) was employed to estimate the population genetic diversity, including the number of different alleles (*N*a), number of effective alleles (*N*e), *H*_O_, and *H*_E_. The within-population inbreeding coefficient *F*_IS_ was estimated with GENEPOP v4.2 (Raymond and Rousset, 1995). Associations among the genetic diversity indices (*H*_O_ and *H*_E_) with population locations (latitude and longitude) were investigated using a simple linear regression analysis.

The inverse distance weighted (IDW) interpolation function implemented in the Geographic Information System (GIS) software ArcGIS 9.3 (ESRI, Redlands, CA, United States) was used to estimate the geographic patterns of *H*_O_ and *H*_E_ for all 29 *Q. fabri* populations.

We also conducted principal coordinates analysis (PCoA) with GENALEX v6.5 (Peakall and Smouse, 2012), where a pairwise, individual-by-individual genetic distance matrix was calculated using the genetic distance option. Analysis of molecular variance (AMOVA) was performed using *R*-statistics in ARLEQUIN v3.11 to infer the genetic differentiation among populations. The significance of fixation indices was tested based on 1,000 permutations for the AMOVA results (Excoffier et al., 2005).

### Genetic Structure and Gene Flow Analysis

The STRUCTURE v2.3.4 software (Pritchard et al., 2000) was used to examine the range-wide genetic structure among populations of *Q. fabri*. Eight independent runs were performed for each *K* from 1 to 15 with 200,000 burn-in steps followed by 500,000 Markov chain Monte Carlo (MCMC) steps. The STRUCTURE output was analyzed and visualized by using Structure Harvester^1^, and the optimal number of clusters (best *K*) was estimated according to Pritchard et al. (2000) and Evanno et al. (2005). Graphics were displayed with the DISTRUCT program (Rosenberg, 2004).

In order to infer historical gene flow (*N*m) patterns, MIGRATE-N v3.6 (Beerli, 2006) was used to estimate the effective population sizes (θ) and mutation scaled immigration (*M*) among the groups identified by STRUCTURE. In this analysis, we used a maximum likelihood procedure and a Brownian motion microsatellite model with 10 short chains following 5,000 steps and three long chains of 50,000 iterations. We sampled every 100 steps under a constant mutation model and discarded the first 10,000 records as a burn-in.

### Approximate Bayesian Computation (ABC) of Species Demography

Based on the inferred STRUCTURE result (optimal *K* = 2, Supplementary Figure 1), we proposed a hierarchical population structure for the three population groups of *Q. fabri* according to the following steps. For *K* = 2, we checked the *q*-value for inferred ancestry in the data set for each individual in the two genetic clusters, and samples where *q* ≥ 0.9 and *q* < 0.9 were regarded as genetically pure and introgressed individuals, respectively (Lepais et al., 2009; Peñaloza-Ramírez et al., 2010; Besnard et al., 2014; Tsuda et al., 2015). We then calculated the proportion of pure individuals (Qp) for each collected population and employed a threshold of 0.80 to partition *Q. fabri* populations, where those with Qp-values ≥ 0.8 were treated as relatively “pure” populations, and populations with Qp-values < 0.8 were regarded as “admixed” populations. Given this threshold, the 29 oak populations were partitioned into three population groups comprising two “pure” population groups and an “admixed” population group for demographic simulation with the ABC in DIYABC v2.0.4 (Cornuet et al., 2014).

Eight alternative demographic history scenarios were simulated for the three *Q. fabri* population groups (Supplementary Table 2 and Supplementary Figure 2). Scenario 1: three population groups diverged from a common ancestral population at time (ta). Scenario 2 and Scenario 3: hierarchical divergence models where population group 2 merged with group 1 (or group 3) at t1 and then group 3 with group 1 at ta. Scenario 4 and Scenario 5: hierarchical divergence models where admixed population group 2 merged with group 1 (or group 3) at t1 and then group 3 (or group 1) with the admixed group (group 2) at ta. Scenario 6: isolation with admixture model where population group 2 was considered to be a genetic admixture, and this group was hypothesized to be the admixture of group 1 and group 3 at t1, and the two pure groups (groups 1 and 3) then merged at ta. Scenario 7 and Scenario 8: mysterious group 4 (referred to as the ghost ancestor) was assumed to have existed, where admixed group 2 was considered to be the admixture of group 1 (or group 3) and mysterious group 4 at t1, and group 3 then merged with group 1 and mysterious group 4 at ta.

We performed 100,000 simulations for each scenario in DIY-ABC. To identify the most likely model, we selected 1% of the stimulated data sets closest to the observed data by logistic regression and estimated the relative posterior probability (PP) with 95% confidence intervals (95% CI) for each scenario. In order to estimate type I and type II errors in the power of model selection, we simulated 500 pseudo-observed data sets for the plausible scenarios. For the best supported scenario, we estimated the posterior distributions of all parameters and evaluating the relative median of absolute errors (RMAE) using 500 pseudo-observed data sets from the simulated data sets. We also estimated the goodness of the fit for 1,000 simulated pseudo-observed data sets by principal component analysis (PCA) (Cornuet et al., 2010) for model assessment.

### Isolation-by-Distance and Isolation-by-Environment Analyses

We analyzed the isolation-by-distance (IBD) and isolation-by-environment (IBE) patterns among *Q. fabri* populations with Mantel tests to evaluate the correlations between genetic and geographical/ecological distances. To evaluate the ecological distance, we selected six bioclimatic factors (we focused on the temperature and precipitation because these two factors have important effects on growth and flowering in tree species) that contributed to the phylogenetic patterns and geographic distribution of *Quercus* species (Xu et al., 2013, 2016; Qian and Sandel, 2017; Yang et al., 2018): annual mean temperature (BIO1), temperature seasonality (BIO4), mean temperature of coldest quarter (BIO11), annual precipitation (BIO12), precipitation seasonality (BIO15), and precipitation of the driest quarter (BIO17). Climatic variables at 2.5 arc-min resolution were obtained from the WorldClim version 2^2^. The pairwise population *F*_ST_ distance was generated as the genetic distance with ARLEQUIN v3.11 (Excoffier et al., 2005). The geographical distance and bioclimatic distance matrices were estimated using the Euclidean method in PASSaGE v2 (Rosenberg and Anderson, 2011). Correlations among distance matrices were then estimated using a two-tailed Mantel test with 999 permutations. After detecting a significant correlation between the distances for geographical and bioclimatic factors (*r* = 0.637, *p* = 0.001), we also performed a partial Mantel test between the genetic differentiation and geographical distances/bioclimatic factors where the bioclimatic or geographical matrix was controlled individually. These Mantel tests were implemented in PASSaGE v2.

Redundancy analysis (RDA) and partial RDA were performed using the R package “vegan” (Oksanen et al., 2018) to test the effect of geography and climate factors on the genetic variations. In total, 200 allele frequencies were obtained as response variables according to the methods described by Li et al. (2019). The principal coordinates of neighbors matrices (PCNMs) were obtained as geographic variables from a truncated matrix comprising the great circle distances between sample populations with the *pcnm* function in the R package “vegan” and the *distVincentyEllipsoid* function in the R package “geosphere” (Hijmans, 2017). We selected equal numbers of geographic variables and climate variable to avoid bias for the IBD and IBE analyses. Finally, a total of 12 predictors (Supplementary Table 3) were applied in the full RDA model. Partial RDA was conducted for the geographic variables with the climatic effects controlled and for the climatic variables with the geographic effects controlled in order to determine the proportions of genetic variation explained by geographic and climatic variables. Model significance was tested after 999 permutations.

### Niche Analyses on Environment Space

Ecological niche comparisons were performed to test whether the different climatic niches contributed to the population differentiation. First, a PCA (hereafter standard PCA) was conducted to examine the climatic variability in the actual niches of the genetic populations across the total climatic space. Second, we estimated the partial environment space (*E*-space) using PCA-env approach developed by Broennimann et al. (2012), where we employed the R scripts provided by Herrando-Moraira et al. (2019). The ecological backgrounds of the three defined population groups (group 1, group 2, and group 3 in the ABC simulation) were selected from a minimum convex polygon with a buffer size of 0.3 degrees, as recently proposed by Silva et al. (2016). The values of the six climatic factors were extracted from the background to construct the available environmental space for the two principal axes, and a kernel smoothing method was utilized to calculate the density of population occurrences. For the new dimensional surface, multiple range PCA-env plots were obtained representing all available climates and occupied conditions simultaneously for occurrence densities of 20 and 100%. Niche overlaps were evaluated using Schoener’s *D* metric, which ranges from 0 (no overlap) to 1 (complete overlap) (Schoener, 1970).

## Results

### Microsatellite Genotyping and Genetic Diversity

**FIGURE 2:**
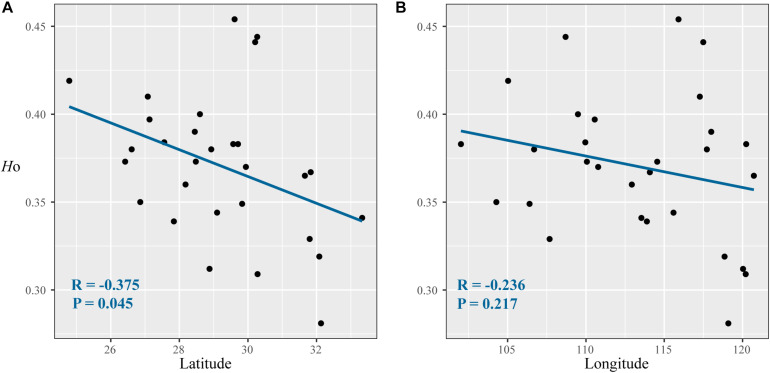
Correlations between diversity parameters (*H*_O_) with latitude **(A)** and longitude **(B)** at 17 nuclear microsatellite loci for *Quercus fabri* populations.

After Bonferroni correction, four SSR loci (FIR004, PIE102, PIE267, and QrZAG7) deviated significantly from Hardy–Weinberg equilibrium in most populations due to the deficiency of homozygotes in these populations. In total, 17 SSR loci yielded 217 alleles in 490 *Q. fabri* individuals, and the other genetic diversity parameters are summarized in Supplementary Table 4. Estimates of genetic diversity showed that these populations had higher values than the means in all populations, e.g., LC, BJ, LL, GY, HH, ST, and SW (*H*_O_: ranged from 0.380 to 0.444; *H*_E_: ranged from 0.547 to 0.609) (Table 1). IDW analysis suggested that these populations were mostly located in mountainous regions (e.g., Yunnan–Guizhou Plateau, LL; Xuefeng Mountain and Wuling Mountain, LC, BJ, HH, DK; Wuyi Mountains and Tianmu Mountain, SW and ST) (Supplementary Figure 3). Correlation analysis indicated a marginal significant decline in *H*_O_ along increasing latitudes (*R* = −0.375, *p* = 0.045), but the variations in *H*_O_ were not significantly correlated with longitude in the species distribution (*p* = 0.217) (Figure 2). In addition, non-significant linear correlations were determined between latitude/longitude and *H*_E_ (latitude: *R* = −0.009, *p* = 0.964; longitude: *R* = −0.264, *p* = 0.167).

### Genetic Variation and Population Structure

AMOVA detected significant genetic differentiation among the three groups (*R*_*CT*_ = 0.10, *p* < 0.001), but only 9.56% of the genetic variation was partitioned. At the population level, 16.30% of the variation was partitioned among populations and 83.70% within populations (*R*_ST_ = 0.16, *p* < 0.001) (Table 2). Bayesian analysis of the population structure suggested an optimal structure of two genetic clusters (*K* = 2) among *Q. fabri* populations based on delta *K* simulations (Supplementary Figure 1). At *K* = 2, all *Q. fabri* populations were assigned to two genetic clusters, including the “blue” and “orange” genetic clusters (Figure 1A). According to the threshold (Qp-value) employed in our ABC simulations, the 29 oak populations were classified into three population groups corresponding to the STRUCTURE results: five populations (LD–WG shown in “blue”) were identified as “genetically pure” population group 1, mainly located in western and central China, and WG was concentrated at the eastern edge of the Qinling Mountains; 18 populations (HH–JCS shown in “orange”) were located in eastern and middle China at relatively low altitudes, and they were identified as “genetically pure” population group 3; and six populations (BJ–JY) belonging to “admixed” population group 2 were located in the contact regions (Figure 1A). Similarly, PCoA suggested that individuals in the two genetic clusters were mostly separated, but individuals from mixed population group 2 overlapped with samples from the two “genetically pure” population groups (Figure 1B).

**TABLE 2:** Analysis of molecular variance (AMOVA) based on SSR data among three groups and all populations of *Quercus fabri.*

Source of variation	*df*	SS	Variation (%)	*R*-statistics
**Three groups**				
Among groups	2	6384.41	9.56	*R*CT = 0.10***
Among populations within groups	26	10976.94	9.98	*R*SC = 0.11***
Within populations	951	77,923.41	80.45	*R*ST = 0.20***
**Species**				
Among populations	28	17,361.35	16.30	*R*ST = 0.16***
Within populations	951	77,923.41	83.70	

df, degrees of freedom; SS, sum of squares. Significance levels: ***p < 0.001; 1,000 permutations.

### Demographic History

ABC analyses showed that scenario 6 had the highest PP and 95% CI compared with other scenarios (Supplementary Table 5 and Supplementary Figure 4). Analyses conducted to estimate the confidence of the scenario selection based on 500 pseudo-observed data sets indicated that type I and type II errors were low for the best supported scenario (Supplementary Table 5). The RMAE values for most of the parameters were also low (<0.20) in most cases, thereby indicating the reliability of the posterior parameter estimates (Supplementary Table 6 and Supplementary Figures 5, 6). The best demographic model (scenario 6) indicated that the two “genetically pure” population groups (groups 1 and 3) initially split from the most recently common ancestor of *Q. fabri* and then formed the “genetically mixed” population group (group 2) *via* genetic admixture between population groups 1 and 3. The median values of the effective population sizes for N1, N2, N3, and NA were estimated as 7.83 × 10^4^, 6.19 × 10^4^, 8.85 × 10^4^, and 3.11 × 10^3^, respectively, thereby suggesting that the effective population sizes of the current population groups underwent expansion after intraspecific divergence (Supplementary Table 6). However, the effective population size for N3 (8.85 × 10^4^, 90% CI = 6.48 × 10^4^–9.80 × 10^4^) presented here should be treated with caution because the estimated value is approaching the maximum threshold of prior setting (Supplementary Table 2). In addition, we had tested the models with increased prior parameter settings, but the PCA evaluation showed unreliable results for these alternatives (Supplementary Note 2). Therefore, the parameter settings were retained as in Supplementary Table 2 and PCA presented in Supplementary Figure 6 seems more reasonable. Besides, the admixture rates (r) were detected between Pop1/Pop3 and Pop2 as: Pop1→Pop2 = 0.449, Pop3→Pop2 = 0.551 (Supplementary Table 6). The estimated median times of divergence between group 1 and group 3 (ta), and group 2 generated by the genetic admixture of group 1 and group 3 (t1) were 4.99 × 10^4^ (90% CI = 1.53–8.29 × 10^4^) and 6.72 × 10^2^ (90% CI = 1.02 × 10^2^–2.85 × 10^3^) generations ago, respectively (Supplementary Table 6). If the generation time is assumed to be 80 years for *Q. fabri*, then the divergence times for ta and t1 corresponded to 3.99 Ma (90% CI = 1.22–6.63 Ma) and 53.76 ka (90% CI = 8.16 ka–228 ka), respectively.

MIGRATE analyses showed that asymmetrical historical gene flow might have occurred among population groups 1 and 3, where the estimated migration rate from group 1 to group 3 was higher than the reverse (*M*_1→3_ = 11.77 vs. *M*_3→1_ = 4.28). For the admixed population group (group 2), the estimated gene flow from group 1 and group 3 into group 2 was greater than in the opposite directions, respectively (*M*_1→2_ = 8.45 vs. *M*_2→1_ = 5.26; *M*_3→2_ = 11.24 vs. *M*_2→3_ = 7.37) (Table 3). The estimated population sizes (and 90% CI) were 5.02 (4.84–5.23) for group 1, 2.01 (1.90–2.10) for group 2, and 2.09 (2.03–2.15) for group 3 (Table 3).

**TABLE 3:** Mutation-scaled effective population size (θ) and mutation-scaled effective immigration rate (*M*) for three groups of *Quercus fabri* estimated using MIGRATE-N.

Migrate	θ	*M* (*m*/μ)		
		
		Group 1→	Group 2→	Group 3→
**Group 1**	5.02 (4.84–5.23)	–	5.26 (5.05–5.48)	4.28 (4.09–4.48)
**Group 2**	2.01 (1.90–2.10)	8.45 (8.07–8.85)	–	11.24 (10.77–11.99)
**Group 3**	2.09 (2.03–2.15)	11.77 (11.37–12.18)	7.37 (7.05–7.69)	–

*Ninety percent confidence intervals are presented in parentheses; θ, 4Neμ; →, source populations; μ, mutation rate.*

### Tests of Isolation by Distance and Isolation by Environment

The Mantel tests indicated that both IBD and IBE had significant effects on the genetic divergence of *Q. fabri*, but the effect of IBE was greater than that of IBD (*R*_D_ = 0.4445, *p* = 0.001; *R*_E_ = 0.6359, *p* = 0.001) (Supplementary Table 7). Given the significant association between geographic distance and ecological distance for *Q. fabri*, partial Mantel tests were performed and they indicated a significant IBE pattern for all populations (*R*_E_ = 0.5109, *p* = 0.003), whereas IBD was not significant (*R*_D_ = 0.0662, *p* = 0.378). For the defined population groups, Mantel tests showed that both IBD and IBE had no significant effects on the genetic divergence of group 1 and group 2. By contrast, significant IBD was observed for population group 3 (*R*_D_ = 0.4007, *p* = 0.002) (Supplementary Table 7).

**FIGURE 3:**
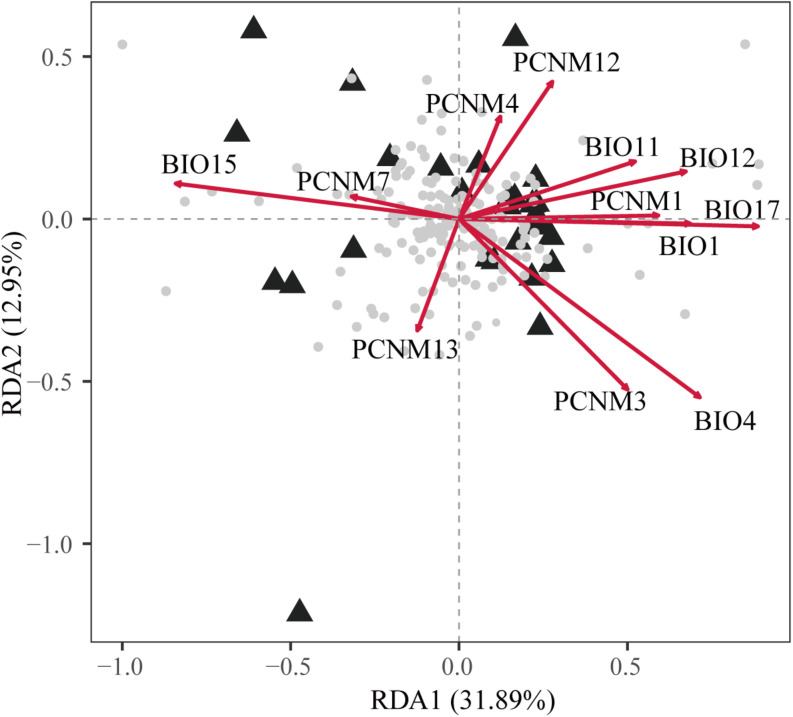
Biplot obtained by redundancy analysis (RDA) showing the associations between genetic variations at 17 nuclear microsatellite loci with geographic and climatic variables. The gray dots at the center represent the allelic variables. The principal coordinates of neighbor matrices (PCNMs) were used as geographic variables comprising PCNM1, PCNM3, PCNM4, PCNM7, PCNM12, and PCNM13 and six climatic variables comprising BIO1, BIO4, BIO11, BIO12, BIO15, and BIO17. The proportion of the total genetic variation explained by each axis is shown in parentheses.

The full RDA model (*df* = 12, *F* = 1.761, *R*_adj_^2^ = 0.246, *P* = 0.001) and both partial RDA models were significant with respect to climate (*df* = 6, *F* = 1.344, *R*_adj_^2^ = 0.071, *P* = 0.007) and geography (*df* = 6, *F* = 1.413, *R*_adj_^2^ = 0.085, *P* = 0.002). The full model explained 56.91% of the total variation and partial RDA demonstrated that climate and geography explained 21.71% and 22.84% (Supplementary Table 8), respectively. About 12.36% of the overall genetic variation was explained by the collinearity between geographic and climatic variables. In the full model, five spatial descriptors and one climatic climate predictors were significant (Supplementary Table 3). Among all of the variables, BIO17 (precipitation of driest quarter) BIO4 (temperature seasonality) and PCNM4 had the highest scores on the first three axes (Figure 3 and Supplementary Table 3).

### Niche Analyses Based on Environment Space

**FIGURE 4:**
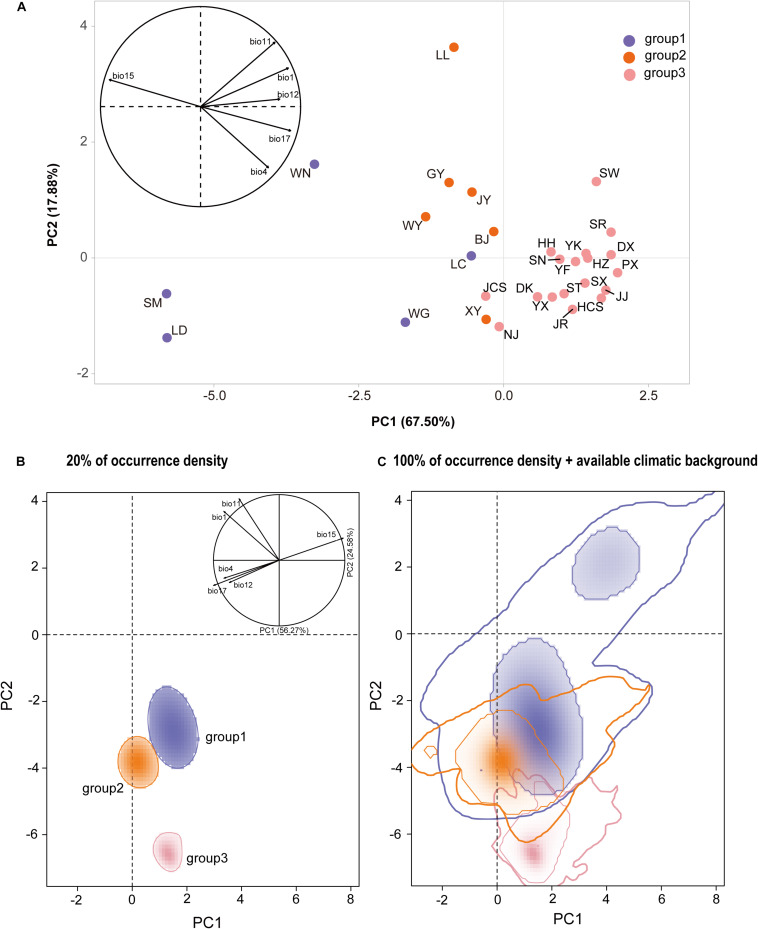
**(A)** Standard principal component analysis (PCA) performed with climatic values for *Quercus fabri* populations. **(B)** Climatic space constructed over all background areas and actual niches of all populations in this study, where the solid line represents an occurrence density of 20%, and **(C)** an occurrence density of 100% is denoted by the thin line and 100% of available climatic background by the thick line.

According to standard PCA, the first three principal components accounted for 96.41% of the total climatic variation (PC1 = 67.50%, PC2 = 17.88%, and PC3 = 11.03%, Supplementary Table 9 and Figure 4A). The first principal component (PC1) was mainly explained by BIO15 (precipitation seasonality), the second (PC2) by the BIO11 (mean temperature in the coldest quarter), and the third (PC3) by BIO12 (annual precipitation) (Supplementary Table 9 and Figure 4A). The niche distribution of group 2 in the climatic PCA space partially overlapped with those of group 1 and group 3. Group 1 had a wider climatic niche distribution than group 2 and group 3 (Figure 4A).

According to PCA-env analysis, the first two axes explained 80.85% of the total variation in the climatic conditions across the population distributions of *Q. fabri* (PC1 = 56.27% and PC2 = 24.58%), where the first component (PC1) was mainly explained by BIO1 (annual mean temperature) and the second by BIO11 (mean temperature of coldest quarter) (Supplementary Figure 7). Multiple niche plots for the occurrence density of 20% (Figure 4B) showed that group 2 was clearly separated from population group 1 and group 3. When the occurrence density of 100% was plotted in the PCA-env space, partial overlaps in climatic space were detected among all the three groups (Figure 4C). Group 1 and group 3 shared their climate niche to a low degree, whereas high overlapping was found for group 1 and group 2. In addition, the overlap *D* values between the groups compared were generally low (group 1 with group 3, *D* = 0.02; group 1 with group 2, *D* = 0.07; group 2 with group 3, *D* = 0.02).

## Discussion

### Environmental Effects on Genetic Diversity

Genetic diversity is a consequence of long-term historical evolution in populations (Zhang et al., 2019). Our genetic survey of *Q. fabri* revealed several distinctive features relative to reported patterns for other tree species distributed in subtropical China. We detected moderate nSSR gene diversity (17 nSSRs, *H*_E_ = 0.542), and it was higher than the nuclear gene diversity of *Populus adenopoda* (10 nSSRs, *H*_E_ = 0.418; Fan et al., 2018) and *Loropetalum chinense* [amplified fragment length polymorphism (AFLP), *H*_E_ = 0.263] (Gong et al., 2016) but lower than those in *Q. acutissima* (12 nSSRs, *H*_E_ = 0.760; Zhang et al., 2013). In addition, the populations located in mountainous areas (e.g., Wuyi Mountain, Wuling, Tianmu, and Yungui plateau) exhibited higher genetic diversity than those located in lowlands (Supplementary Figure 3). It is possible that these mountains provided a diverse range of thermal, hydric, and edaphic conditions, as well as complex and mesic habitats for *Q. fabri* during glacial periods in the Pleistocene. Thus, mountains are likely to have served as important refugia for *Q. fabri* in a similar manner to many other plants, according to previous studies (e.g., Gavin et al., 2014; Tian et al., 2015; Zhang et al., 2019).

### Ancient Divergence and Admixture Triggered by Heterogeneous Environments

Genetic structure patterns are produced by evolutionary and demographic processes at different temporal scales (Morris et al., 2008). In the present study, STRUCTURE analysis indicated the existence of two genetic structures (*K* = 2; Figure 1A) that roughly corresponded to western and eastern China. In addition, ABC analyses suggested that the divergence time among groups (group 1 and group 3) could have occurred in the late Pliocene (*ca.* 3.99 Ma, 1.22–6.63 Ma). The differentiation time was similar to previous results obtained based on three chloroplast DNA regions (*ca.* 2.73 Ma, 1.15–5.21 Ma) (Chen et al., 2020). Compared with other deciduous oak species in China, this initial divergence time is similar to that for *Q. acutissima* (2.96 Ma) (Zhang et al., 2018) but earlier than those for *Q. variabilis* (1.45 Ma) (Chen et al., 2012) and *Q. mongolica* (1.38 Ma) (Zeng et al., 2015).

In the present study, the margins of the eastern and western populations were determined along the boundary of the second and third Chinese steppe regions, which correspond to the eastern margin of Yunnan–Guizhou Plateau (Committee of Chinese Academy of Sciences for Physical Geography of China, 1985). The accelerated uplift of the QTP during the period from 3.4 to 2.6 Ma leads to an increase in the activity in the adjacent areas (Li and Fang, 1999; Liu et al., 2013), such as the uplift of the west Qinling Mountain and formation of fault basins on the Yunnan–Guizhou Plateau (An et al., 1999). Moreover, the rise of the QTP dramatically modified the global and East Asian climate by triggering and intensifying the Asian monsoon. In addition, Wuyi and Naling Mountains gradually uplifted in the late Pleistocene (Chen and Zhou, 1993; Luo et al., 2010). These physical features could have reduced the gene flow among different regions. In particular, the great difference in topography (average altitude, second step: 1,260 m; third step: 251 m) had major effects on environmental factors (Yang et al., 1989; Qi et al., 2017). An obvious climatic difference occurs between west and east China, with a moist maritime climate in the east but a seasonal inland climate in west China. The precipitation is generally low in the west but higher in the east (Li et al., 2014; Gao et al., 2020). Therefore, the variable climate conditions and heterogeneous habitats caused by regional geological events drove the isolation of the western and eastern lineages. A similar west–east pattern has been found in the EBLF in subtropical China, such as for *Castanopsis fargesii* (Sun et al., 2014), *Castanopsis eyrei* (Shi et al., 2014), and *Castanopsis carlesii* (Sun et al., 2016). Our investigation of *Q. fabri* also suggests that the boundary between the second and third Chinese steppes played an important role in shaping the genetic structure of deciduous tree species in subtropical China. ABC and MIGRATE analyses both provided evidence of intraspecific bidirectional and asymmetric gene flow among the three defined population groups (Table 3 and Supplementary Table 6). These heterogeneous environments actually provided the necessary preconditions for the adaptive divergence of fragmented populations (e.g., Liu et al., 2013). Moreover, the effects of geographical isolation and environmental heterogeneity on the genetic patterns of *Quercus* species, have been reported in previous studies for example, *Quercus engelmannii* (Ortego et al., 2012), *Quercus chrysolepis* (Ortego et al., 2015), *Quercus kerrii* (Jiang et al., 2018), and *Quercus acutissima* (Gao et al., 2020).

Our analysis detected a recent intraspecific admixture event in *Q. fabri* between group 1 and group 3, which was estimated to have occurred during the following 53.76 ka (90% CI 8.16–228 ka) (Supplementary Table 6) when the late Pleistocene glacial–interglacial cycles comprised the dominant climatic regime, i.e., the population expansion during interglacial periods would provide opportunities for population contacts in different regions (Mayol et al., 2015). Expansion of *Q. fabri* during the interglaciation was further supported by the previous ENM analysis (Chen et al., 2020). Paleoclimatological studies suggested that the last glacial interglacial (LGI, 75–100 ka, MIS 5; Cui et al., 2011) had a climate as warm as or warmer than today (Kukla et al., 2002). Therefore, *Q. fabri* populations could have migrated from the different regions to track their optimal ecological conditions during the warm periods and expand to larger areas of suitable habitat, which triggered the occurrences of genetic admixture. Overall, our results indicate that the geography and climate jointly shaped the genetic structure of *Q. fabri*.

### Ecological Variation

Ecological processes play an important role in species differentiation (Gao et al., 2020). Ecological niche partitioning would have reinforced the divergence of the lineages following their initial spatial isolation, and it may have led to some degree of differential adaptation to varying environmental conditions (Liu et al., 2013). Thus, it is plausible that the current distribution of *Q. fabri* is primarily determined by the QTP uplift (∼3.4–2.6 Ma), which is proposed to have associated with past geographical and climatic fluctuations. Our niche models produced based on the Broennimann method (Broennimann et al., 2012) suggested that the three defined population groups of *Q. fabri* might have occupied different climate niches (Figure 4B), although some niche overlaps may have occurred among lineages according to simulation with 100% occurrence (Figure 4C). In addition, the standard PCAs provided evidence for the roles of ecological factors in triggering population differentiation in *Q. fabri* (Figure 4A). *E*-space analyses suggested that the annual mean temperature (BIO1), mean temperature of coldest quarter (BIO11), and precipitation seasonality (BIO15) were potential ecological factors associated with intraspecific differentiation (Supplementary Figure 7 and Figures 4B,C). The temperature seasonality (BIO4) also contributed significantly to the geographic patterns of climatically genetic variation (Figure 3 and Supplementary Table 3). Climate seasonality (e.g., temperature and precipitation seasonality) is among the most important ecological factors that affect phenology (e.g., flowering time and growing season) (Quintero et al., 2014). Thus, it is possible that the gene flow between populations in group 1 and group 3 might have been limited due to potential genetic barriers caused by asynchronous phenology, thereby resulting in population differentiation. Further research based on sufficient genomic data related to environmental selection and phenological observations is necessary to improve our understanding of divergent adaptation and environment isolation processes during *Q. fabri* population differentiation. The contributions of environmental variables to the genetic differentiation in *Q. fabri* were also supported by the IBE and IBD analyses. It should be noted that the spatial Mantel tests did not detect the IBE patterns within groups to a large degree, and thus the genetic structure within groups might have been less correlated with the ecological variables. However, our results suggest that the distribution of *Q. fabri* populations in different regions was mediated by the perturbation of abiotic environment factors, which might have influenced the genetic variation and population differentiation.

## Conclusion

In this study, we used genetic methods and niche analyses to investigate the spatial genetic structure and demographic history of *Q. fabri*, which is an endemic tree species in subtropical China. Our genetic results suggest that the observed patterns of genetic variation and structure among populations of *Q. fabri* could be explained by a combination of both geological and climatic events in the late Pliocene/Quaternary. In addition, ecological analyses indicated clear ecological differentiation among the population structures of *Q. fabri*. Initial topological constraints reinforced by subsequent differential ecological adaptations may have resulted in intraspecific differentiation and the formation of genetic patterns in this oak species. Thus, heterogeneous environmental factors may have affected the spatial genetic variation, structure, and demographic history of this deciduous tree species in subtropical China.

## Data Availability Statement

The original contributions presented in the study are publicly available. This data can be found here: https://datadryad.org/stash/share/NCuKCzVN_yl9MikUBvD8_e41QndMma9daCr6iLe5Z_M.

## Author Contributions

X-DC, JY, and G-FZ designed the research. X-DC and Y-FG performed the experiments. JY, TZ, Y-MZ, XZ, and M-MJ contributed materials and data analysis. X-DC wrote the manuscript. JY, Z-HL, and G-FZ revised the manuscript. All authors contributed to the article and approved the submitted version.

## Conflict of Interest

The authors declare that the research was conducted in the absence of any commercial or financial relationships that could be construed as a potential conflict of interest.

## Footnotes

1 http://taylor0.biology.ucla.edu/structureHarvester/

2 http://www.worldclim.org/

## Supplementary Material

Supplementary Figure 1 Distribution of mean log probability of the data (ln Pr(X| K)) and Delta *K* values are presented for *K* = 1–15 (eight replicates) in populations of *Quercus fabri.*

Supplementary Figure 2 Scenarios tested using approximate Bayesian computation (ABC). In these scenarios, Pop 1, Pop 2, and Pop 3 represent group 1, group 2, and group 3, respectively, t# represents the time in generations, N# denotes the effective population sizes of the different populations, and r# is the immigration rates from donor populations.

Supplementary Figure 3 Inverse distance weighted (IDW) interpolation of observed heterozygosity (*H*_O_) and expected heterozygosity (*H*_E_) for 29 *Quercus fabri* populations (black triangle) in subtropical China.

Supplementary Figure 4 Comparison of posterior probabilities for eight simulated scenarios obtained by logistic regression from 1% of the closest data set in DIYabc.

Supplementary Figure 5 **(A)** Estimate posterior distributions for parameters and **(B)** model checking in DIYabc for the best-supported (scenario 6).

Supplementary Figure 6 Principal component analysis results obtained along the first three axes for the pre-evaluate scenarios and prior distributions in DIYabc.

Supplementary Figure 7 Contribution of each environmental variable to spatial distribution of the PCA-env.

Supplementary Table 1 PCR primers used in this study.

Supplementary Table 2 Prior distributions of the parameters used in DIYabc.

Supplementary Table 3 Scores for the six geographic variables and six climatic variables on the first three RDA axes (RDA1–RDA3) in the full RDA model, and both partial RDA models corresponding to pure geography and pure climate. Significance tests were performed based on 999 permutations for each constrained axis and each constraining variable.

Supplementary Table 4 Genetic diversity parameters estimated at 17 nuclear microsatellite loci in 29 populations.

Supplementary Table 5 Posterior probability and 95% confidence intervals (CI) for each scenarios based on the logistic regression approach for approximate Bayesian computation analyses (ABC). Type I and type II errors are indicated for the best supporting scenarios (in bold).

Supplementary Table 6 Posterior distributions of parameters for the best supported scenario (scenario 6) of the demographic history of *Quercus fabri* with approximate Bayesian computation. Relative median absolute errors (RMAE) based on 500 pseudo-observed data are also indicated for each parameter.

Supplementary Table 7 Results of Mantel tests between genetic (GeD) and geographical (GD) distance, and genetic (GeD) and ecological (ED) distances for *Quercus fabri* and three groups based on nuclear microsatellite data.

Supplementary Table 8 Partitioning of the genetic variation in *Quercus fabri* purely associated with climate and geography using redundancy analysis (RDA) and partial RDA. The proportion of genetic variation that could not be partitioned due to the collinearity between geographic and climatic variables (climate + geography) is also shown.

Supplementary Table 9 Descriptive statistics based on principle components analysis (PCA) of the selected variables. The variables that mainly explained each axis are shown in bold.

Supplementary Note 1 Microsatellite genotype dataset used in this study.

Supplementary Note 2 Principal component analysis results obtained along the first three axes for the pre-evaluate scenarios and prior distributions in DIYabc (based on alternative parameters).
