# Supplementary figures and images for: Spatial Genetic Structure and Demographic History of the Dominant Forest Oak Quercus fabri Hance in Subtropical China
Source: Front Plant Sci. 2021 Feb 4;11:583284. doi: 10.3389/fpls.2020.583284 (PMC7889815; doi:10.3389/fpls.2020.583284)

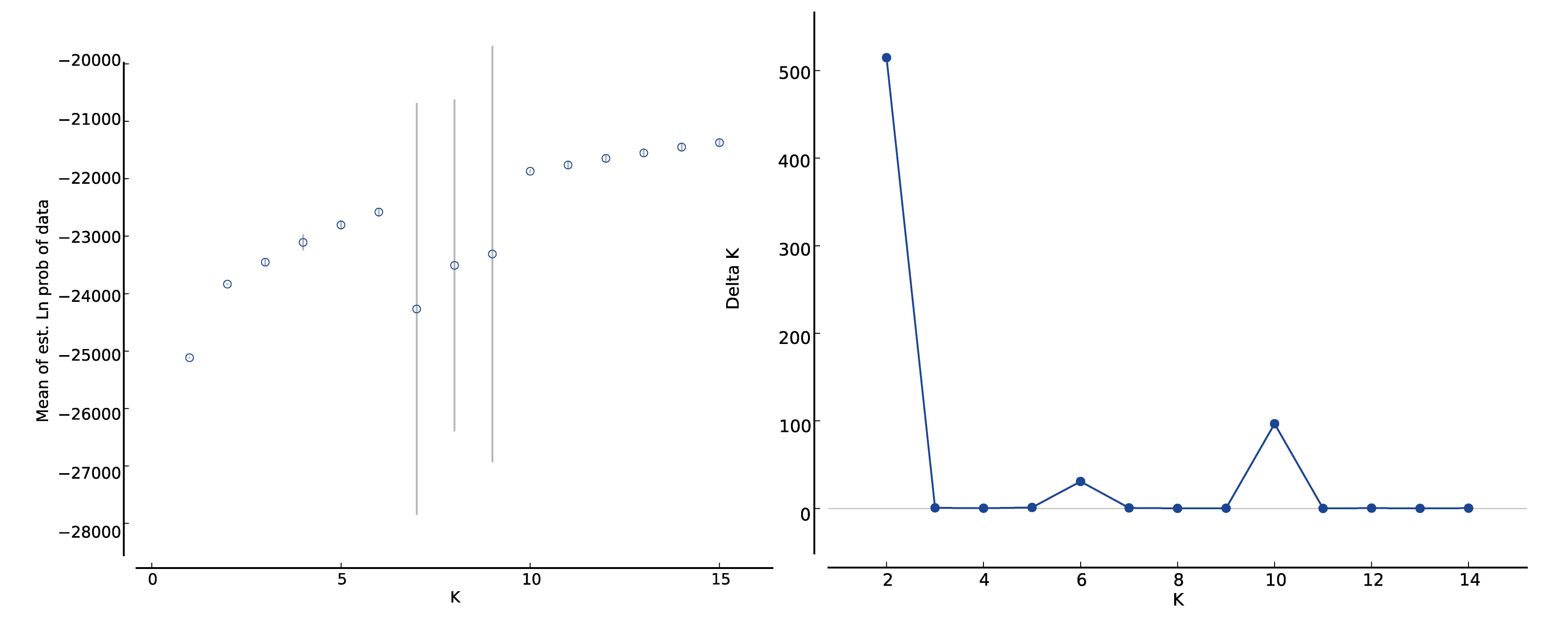

Supplement: Supplementary Figure 1 — Distribution of mean log probability of the data (ln Pr(X| K)) and Delta K values are presented for K = 1–15 (eight replicates) in populations of Quercus fabri. [file Image_1.TIF]

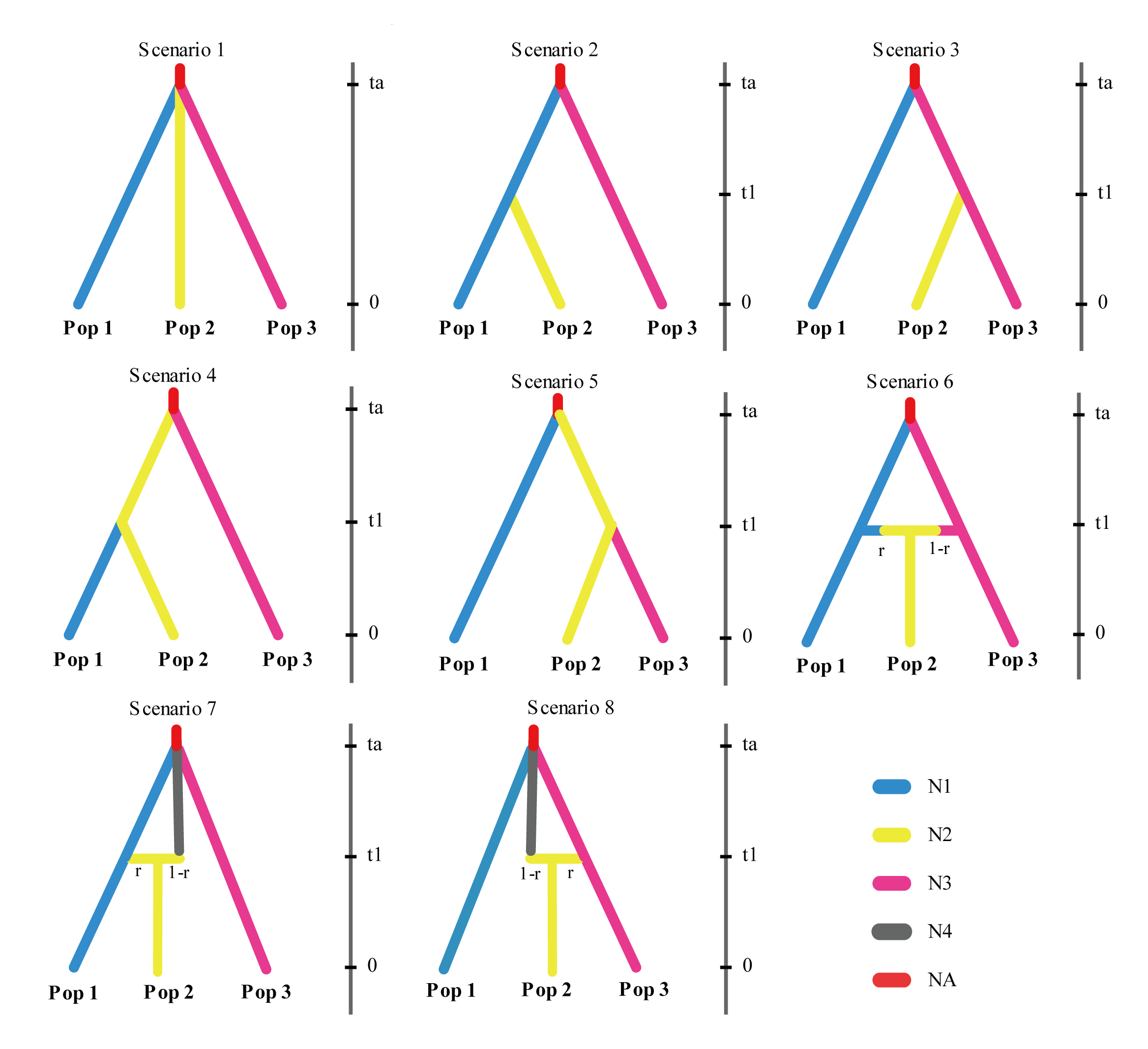

Supplement: Supplementary Figure 2 — Scenarios tested using approximate Bayesian computation (ABC). In these scenarios, Pop 1, Pop 2, and Pop 3 represent group 1, group 2, and group 3, respectively, t# represents the time in generations, N# denotes the effective population sizes of the different populations, and r# is the immigration rates from donor populations. [file Image_2.TIF]

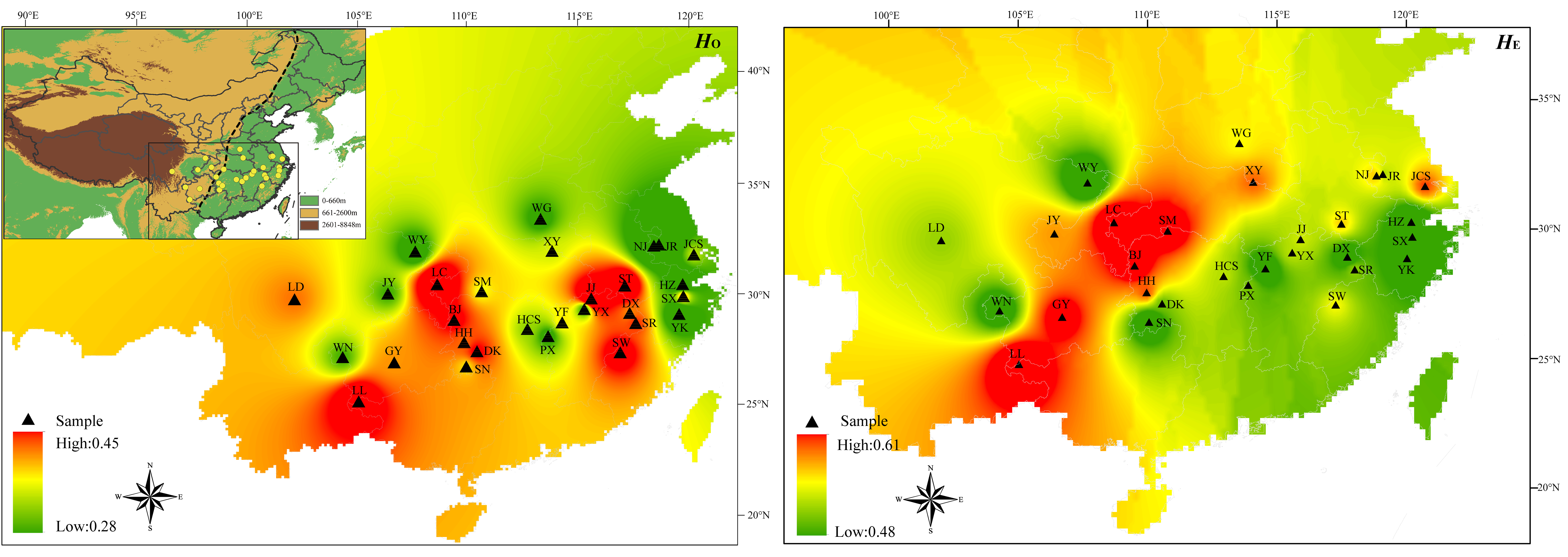

Supplement: Supplementary Figure 3 — Inverse distance weighted (IDW) interpolation of observed heterozygosity (HO) and expected heterozygosity (HE) for 29 Quercus fabri populations (black triangle) in subtropical China. [file Image_3.TIF]

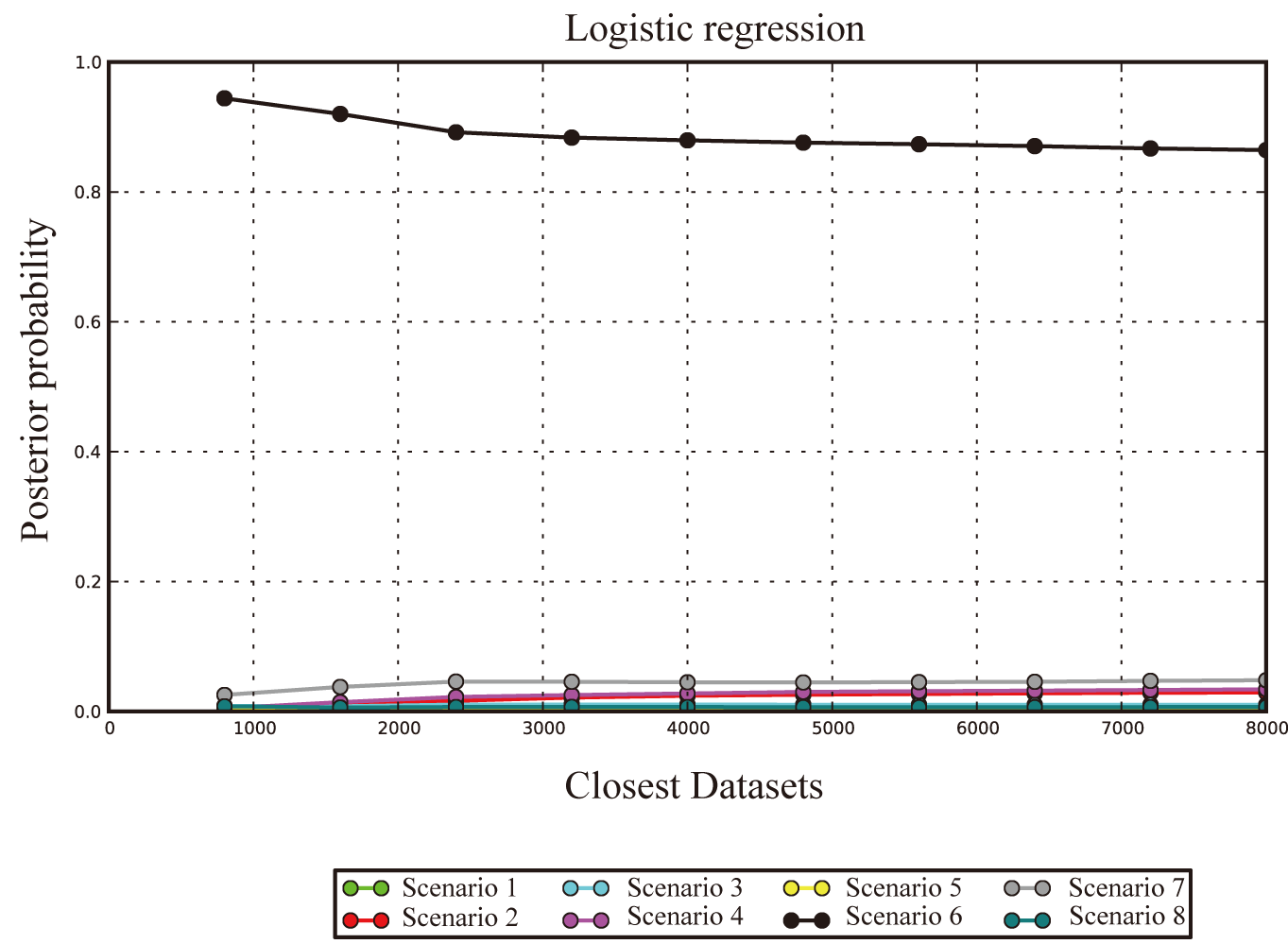

Supplement: Supplementary Figure 4 — Comparison of posterior probabilities for eight simulated scenarios obtained by logistic regression from 1% of the closest data set in DIYabc. [file Image_4.TIF]

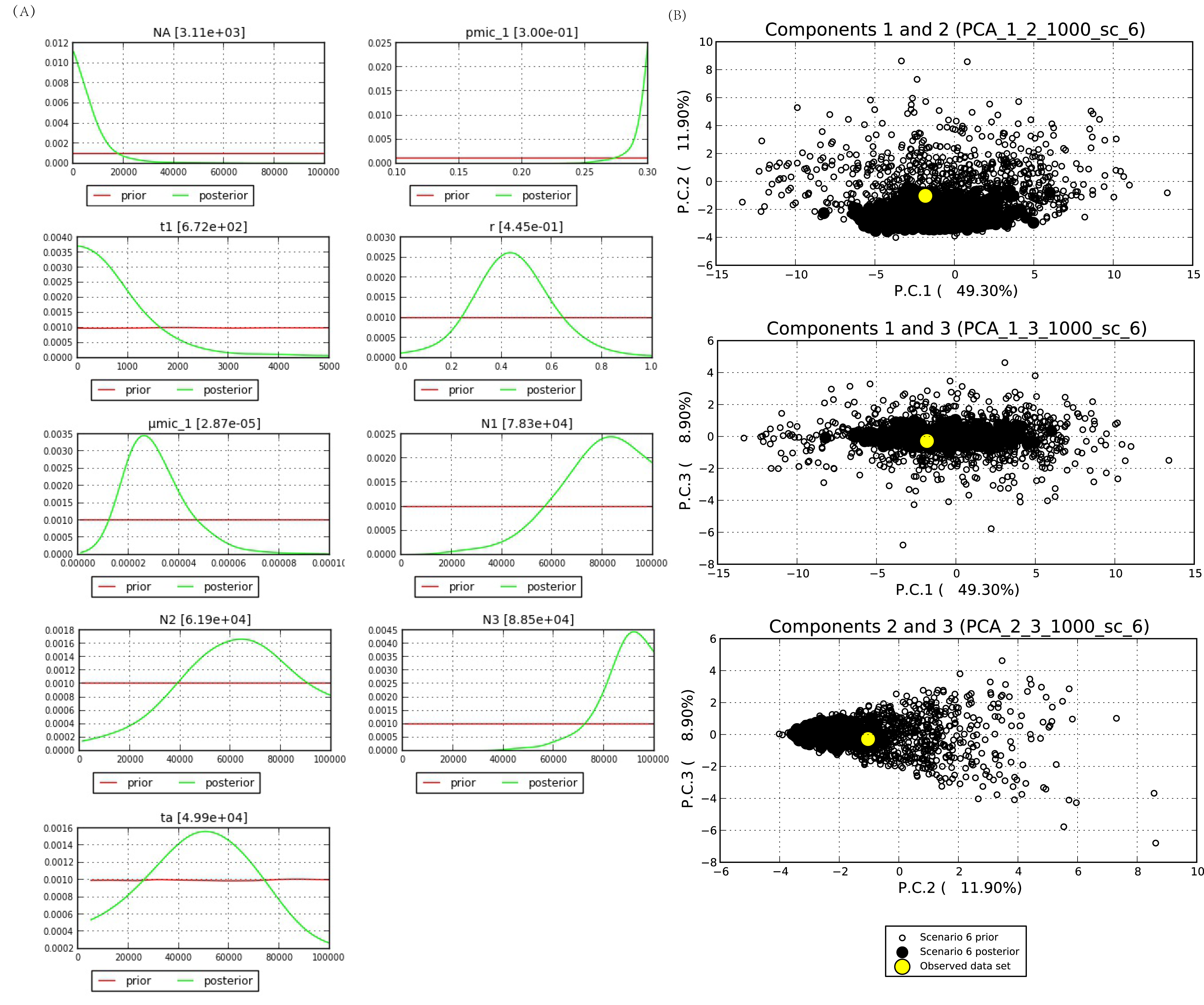

Supplement: Supplementary Figure 5 — (A) Estimate posterior distributions for parameters and (B) model checking in DIYabc for the best-supported (scenario 6). [file Image_5.TIF]

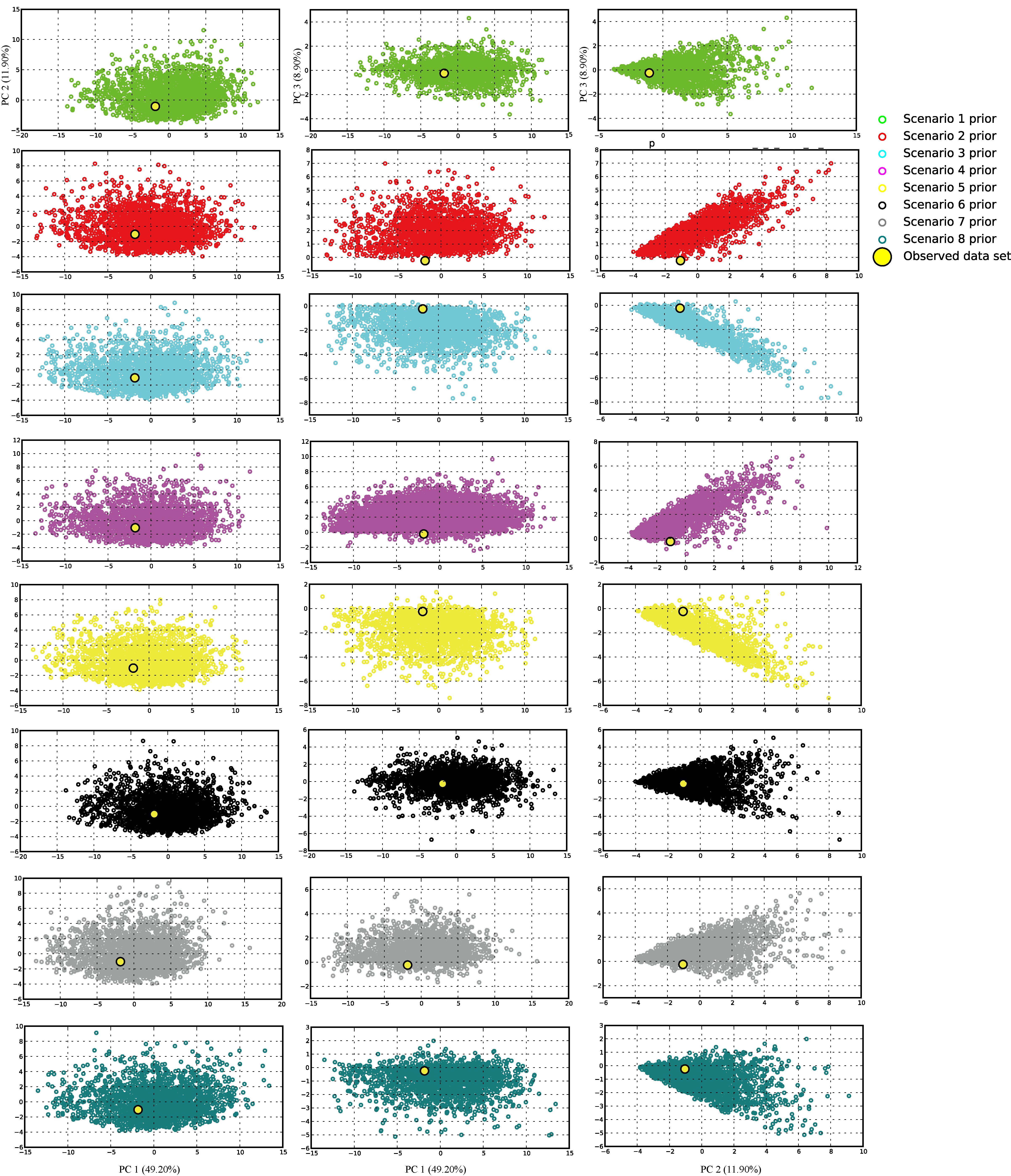

Supplement: Supplementary Figure 6 — Principal component analysis results obtained along the first three axes for the pre-evaluate scenarios and prior distributions in DIYabc. [file Image_6.TIF]

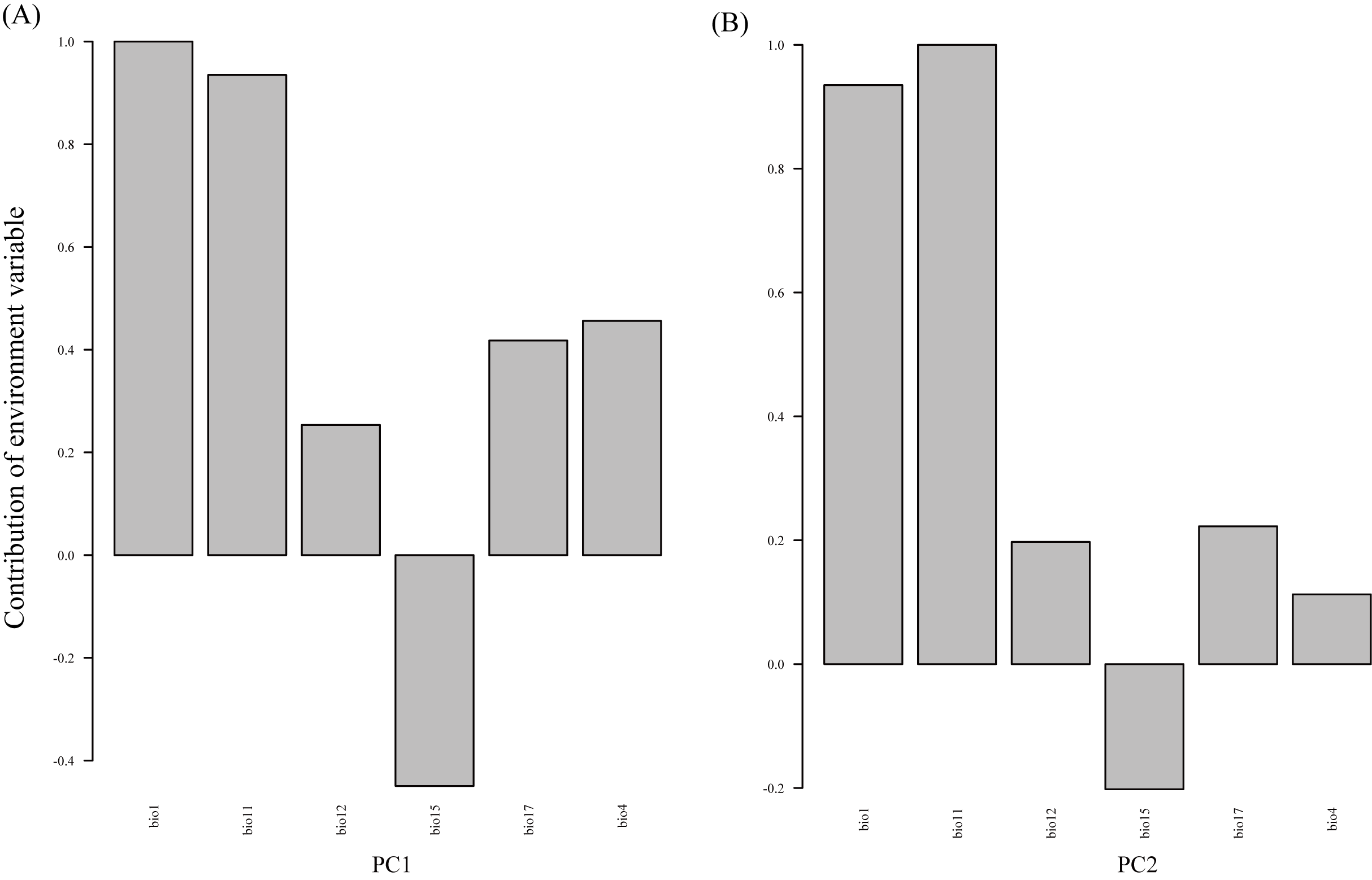

Supplement: Supplementary Figure 7 — Contribution of each environmental variable to spatial distribution of the PCA-env. [file Image_7.TIF]
