# Supplementary material for: Spatial Genetic Structure and Demographic History of the Dominant Forest Oak Quercus fabri Hance in Subtropical China
Source: Front Plant Sci. 2021 Feb 4;11:583284. doi: 10.3389/fpls.2020.583284 (PMC7889815; doi:10.3389/fpls.2020.583284)
Supplement: Supplementary Table 2 — Prior distributions of the parameters used in DIYabc. [file Table_2.docx]

**Table S1** PCR primers used in this study.

| Locus | Primer Sequence(5'–3') | Repeat motif | Tm (◦C) | Reference |
| --- | --- | --- | --- | --- |
| Qv020 | F：CATCGTCGAAGTCGAAGTCA | AAG | 56 | Wang et al. 2015 |
|  | R：CTTCGATTTCACACGCTCAA |  |  |  |
| Qv089 | F：GGGTGAGATTGAAAAGCCAA | AAG/ AGG | 56 | Wang et al. 2015 |
|  | R：GCTTCCTCTTCCGCTTACCT |  |  |  |
| KP855752 | F：GACCCATTGACTGAACTCAT | AG | 56 | Chen and Zhang unpublished data |
|  | R：GTGTCTCTGTCCAAAACTCAA |  |  |  |
| FIR004 | F：TCTCTCTCAGGGCAGCTTCT | CT | 59 | Sullivan et al. 2012 |
|  | R：AACCAAACTCAGATCCAGATTCA |  |  |  |
| KP855993 | F：TATCTTTTGTGGGGGAAGATA | GA | 56 | Chen and Zhang unpublished data |
|  | R：AGAAGGCCTCATCTTTCTTTA |  |  |  |
| KP856046 | F：CAGCTCTGGATGGATATAATG | TC | 56 | Chen and Zhang unpublished data |
|  | R：ACATGCATGGAGAGAAATAGA |  |  |  |
| KP856055 | F：ACGAGAATGACAGGGAAAATA | ACA | 56 | Chen and Zhang unpublished data |
|  | R：TGATCGTCGTGGTAATCTTAG |  |  |  |
| QrZAG112 | F：TTCTTGCTTTGGTGCGCG | GA | 59.3 | Kampfer et al. 1998 |
|  | R：GTGGTCAGAGACTCGGTAAGTATTC |  |  |  |
| PIE227 | F：TACCATGATCTGGGAAGCAAC | TGG | 56 | Durand et al. 2010 |
|  | R：AAGGGCTTGGTTGGGTTAGT |  |  |  |
| PIE267 | F：TCCAACCATCAAGGCCATTAC | AG | 61.6 | Durand et al. 2010 |
|  | R：GTGCGAACAGATCCCTTGTC |  |  |  |
| QrZAG96 | F：CCCAGTCACATCCACTACTGTCC | TC | 60.3 | Kampfer et al. 1998 |
|  | R：GGTTGGGAAAAGGAGATCAGA |  |  |  |
| PIE152 | F：TGTACCTCTTTCCTCTCTCTAAAACT | TA | 50 | Durand et al. 2010 |
|  | R：GAATTTCTAAACCACTAGCATTGAC |  |  |  |
| PIE102 | F：ACCTTCCATGCTCAAAGATG | CT | 61.6 | Durand et al. 2010 |
|  | R：GCTGGTGATACAAGTGTTTGG |  |  |  |
| KP855892 | F：CCTGCTTCCAATATTCTCATA | TTC | 56 | Chen and Zhang unpublished data |
|  | R：GCAAAATCACTCATCAAGAAC |  |  |  |
| MsQ13 | F：ACACTCAGACCCACCATTTTTCC | GA | 60.3 | Dow et al. 1995 |
|  | R：TGGCTGCACCTATGGCTCTTAG |  |  |  |
| QpZAG15 | F：CGATTTGATAATGACACTATGG | AG | 56.4 | Steinkellner et al. 1997 |
|  | R：CATCGACTCATTGTTAAGCAC |  |  |  |
| QrZAG7 | F：CAACTTGGTGTTCGGATCAA | TC | 59.3 | Kampfer et al. 1998 |
|  | R：GTGCATTTCTTTTATAGCATTCAC |  |  |  |

**Reference**

Dow, B.D., Ashley, M.V., and Howe, H.F. (1995). Characterization of highly variable (GA ⁄ CT)n microsatellites in the bur oak, *Quercus macrocarpa.* *Theor. Appl. Genet.* 91, 137–141.

Durand, J., Bodénès, C., Chancerel, E., Frigerio, J.M., Vendramin, G., Sebastiani, F., et al. (2010). A fast and cost-effective approach to develop and map EST-SSR markers: oak as a case study. *BMC Genomics* 11, 570.

Kampfer, S., Lexer, C., Glössl, J., and Steinkellner, H. (1998). Characterization of (GA)n microsatellite loci from *Quercus robur*. *Hereditas* 129, 183–186.

Wang, X., Li, J., Li, Y. (2015). Isolation and characterization of microsatellite markers for an endemic tree in East Asia, *Quercus variabilis* (Fagaceae). *Applications in plant sciences* 3.

Steinkellner, H., Fluch, S., Turetschek, E., Lexer, C., Streiff, R., Kremer, A., et al. (1997). Identification and characterization of (GA ⁄ CT)n microsatellite loci from *Quercus petraea*. *Plant Mol. Biol.* 33, 1093–1096.

Sullivan, A.R., Lind, J.F., McCleary, T.S., Romero-Severson, J., Gailing, O. (2013). Development and characterization of genomic and gene-based microsatellite markers in North American red oak species. *Plant Mol. Biol. Rep.* 31, 231–239.

**Table S2** Prior distributions of the parameters used in DIYabc.

| Parameter | Minimum | Maximum |
| --- | --- | --- |
| Effective population size |  |  |
| N1 | 1000 | 100000 |
| N2 | 1000 | 100000 |
| N3 | 1000 | 100000 |
| NA | 100 | 100000 |
| N4 | 1000 | 100000 |
| Time scale in generations |  |  |
| ta | 5000 | 100000 |
| t1 | 10 | 5000 |
| Mutation model |  |  |
| Mean mutation rate | 1.00E-06 | 1.00E-04 |
| Individual locus mutation rate | 1.00E-07 | 1.00E-03 |
| Mean coefficient P | 1.00E-01 | 3.00E-01 |
| Individual locus coefficient P | 1.00E-02 | 9.00E-01 |
| Mean SNI rate | 0 | 0 |
| Individual locus SNI rate | 0 | 0 |

**Table S3** Scores for the six geographic variables and six climatic variables on the first three RDA axes (RDA1–RDA3) in the full RDA model, and both partial RDA models corresponding to pure geography and pure climate. Significance tests were performed based on 999 permutations for each constrained axis and each constraining variable.

| Variables | Geography and climate | | | |  | Pure geography | | | |  | Pure climate | | | |
| --- | --- | --- | --- | --- | --- | --- | --- | --- | --- | --- | --- | --- | --- | --- |
|  | *P*-value | RDA1*** | RDA2** | RDA3 |  | *P*-value | RDA1* | RDA2 | RDA3 |  | *P*-value | RDA1* | RDA2 | RDA3 |
| PCNM1 | 0.001*** | 0.5916 | 0.01081 | -0.10191 |  | 0.102 | 0.09164 | 0.422160 | 0.31749 |  |  |  |  |  |
| PCNM3 | 0.002** | 0.5004 | -0.52505 | 0.25676 |  | 0.058 | -0.11525 | -0.601628 | -0.06048 |  |  |  |  |  |
| PCNM4 | 0.035* | 0.1225 | 0.31492 | -0.66333 |  | 0.066 | -0.11136 | 0.286129 | -0.36122 |  |  |  |  |  |
| PCNM7 | 0.044* | -0.3189 | 0.06911 | 0.16878 |  | 0.566 | -0.11096 | -0.159184 | 0.05387 |  |  |  |  |  |
| PCNM12 | 0.011* | 0.2769 | 0.42241 | -0.00819 |  | 0.003** | 0.78254 | -0.008305 | -0.43959 |  |  |  |  |  |
| PCNM13 | 0.069 | -0.1238 | -0.34325 | -0.42467 |  | 0.060 | -0.38874 | 0.406238 | -0.02321 |  |  |  |  |  |
| BIO1 | 0.017* | 0.6863 | -0.01432 | -0.13981 |  |  |  |  |  |  | 0.006** | 0.6137 | -0.3408 | 0.35641 |
| BIO4 | 0.225 | 0.7149 | -0.54917 | 0.07495 |  |  |  |  |  |  | 0.175 | 0.1309 | -0.3682 | -0.16643 |
| BIO11 | 0.361 | 0.5225 | 0.17717 | -0.17511 |  |  |  |  |  |  | 0.428 | 0.6387 | -0.2462 | 0.45796 |
| BIO12 | 0.223 | 0.6734 | 0.14633 | 0.06981 |  |  |  |  |  |  | 0.246 | 0.6506 | -0.1138 | 0.03933 |
| BIO15 | 0.055 | -0.8414 | 0.10923 | -0.10621 |  |  |  |  |  |  | 0.037* | -0.5794 | 0.0696 | 0.01679 |
| BIO17 | 0.101 | 0.8873 | -0.02277 | -0.06662 |  |  |  |  |  |  | 0.109 | 0.5295 | -0.1181 | -0.11711 |

BIO1, annual mean temperature; BIO4, temperature seasonality;BIO11, mean temperature of coldest quarter ; BIO12, annual precipitation; BIO15, precipitation seasonality; BIO17, precipitation of driest quarter ;.***, *P* < 0.001; **, *P* < 0.01; *, *P* < 0.05.

**Table S4** Genetic diversity parameters estimated at 17 nuclear microsatellite loci in 29 populations.

| Locus | *Na* | *H*_O_ | *H*_E_ | *A*s | *H*s | *H*_T_ | PIC | *F*_IT_ | *F*_ST_ | *F*_IS_ | *N*m |
| --- | --- | --- | --- | --- | --- | --- | --- | --- | --- | --- | --- |
| Qv020 | 4 | 0.202 | 0.369 | 2.649 | 0.346 | 0.373 | 0.343 | 0.441 | 0.114 | 0.369 | 1.941 |
| Qv089 | 17 | 0.764 | 0.834 | 6.016 | 0.810 | 0.831 | 0.814 | 0.089 | 0.059 | 0.031 | 3.964 |
| KP855752 | 15 | 0.579 | 0.747 | 4.754 | 0.724 | 0.744 | 0.715 | 0.245 | 0.065 | 0.192 | 3.602 |
| FIR004 | 23 | 0.118 | 0.93 | 8.398 | 0.910 | 0.932 | 0.924 | 0.844 | 0.082 | 0.830 | 2.809 |
| KP855993 | 4 | 0.012 | 0.02 | 1.121 | 0.019 | 0.019 | 0.02 | 0.420 | 0.047 | 0.392 | 5.046 |
| KP856046 | 12 | 0.542 | 0.705 | 3.921 | 0.604 | 0.715 | 0.654 | 0.229 | 0.185 | 0.054 | 1.102 |
| KP856055 | 4 | 0.436 | 0.481 | 2.150 | 0.477 | 0.482 | 0.378 | 0.095 | 0.045 | 0.052 | 5.337 |
| QrZAG112 | 7 | 0.245 | 0.289 | 2.423 | 0.225 | 0.267 | 0.275 | 0.145 | 0.184 | -0.048 | 1.109 |
| PIE227 | 8 | 0.449 | 0.621 | 3.836 | 0.489 | 0.611 | 0.581 | 0.276 | 0.228 | 0.062 | 0.847 |
| PIE267 | 12 | 0.277 | 0.784 | 4.952 | 0.685 | 0.775 | 0.752 | 0.644 | 0.162 | 0.576 | 1.298 |
| QrZAG96 | 20 | 0.703 | 0.91 | 7.723 | 0.888 | 0.909 | 0.902 | 0.219 | 0.061 | 0.168 | 3.838 |
| PIE152 | 15 | 0.301 | 0.506 | 3.766 | 0.378 | 0.486 | 0.487 | 0.386 | 0.253 | 0.178 | 0.738 |
| PIE102 | 20 | 0.346 | 0.895 | 7.339 | 0.846 | 0.896 | 0.885 | 0.595 | 0.104 | 0.548 | 2.161 |
| KP855892 | 9 | 0.356 | 0.421 | 3.137 | 0.349 | 0.404 | 0.401 | 0.152 | 0.166 | -0.017 | 1.258 |
| MsQ13 | 14 | 0.315 | 0.633 | 4.455 | 0.536 | 0.624 | 0.608 | 0.524 | 0.182 | 0.418 | 1.127 |
| QpZAG15 | 12 | 0.387 | 0.546 | 3.885 | 0.468 | 0.529 | 0.523 | 0.303 | 0.152 | 0.178 | 1.399 |
| QrZAG7 | 21 | 0.29 | 0.932 | 8.463 | 0.896 | 0.933 | 0.927 | 0.669 | 0.093 | 0.635 | 2.449 |
| All | 217 |  |  |  |  |  |  |  |  |  |  |

**Table S5** Posterior probability and 95% confidence interval (CI) for each scenario based on the logistic regression approach for approximate Bayesian computation analyses (ABC). Type I and type II errors are indicated for the best supporting scenarios (in bold)

| Scenarios | Posterior  Probability | 95% CI | Type Ⅰ | Type Ⅱ |
| --- | --- | --- | --- | --- |
| 1 | 0.0034 | [0.0015,0.0053] |  |  |
| 2 | 0.0293 | [0.0162,0.0425] |  |  |
| 3 | 0.0102 | [0.0054,0.0150] |  |  |
| 4 | 0.0342 | [0.0192,0.0492] |  |  |
| 5 | 0.0031 | [0.0011,0.0051] |  |  |
| **6** | **0.8645** | **[0.8229,0.9061]** | **0.062** | **0.043** |
| 7 | 0.0482 | [0.0286,0.0678] |  |  |
| 8 | 0.0071 | [0.0039,0.0103] |  |  |

**Table S6** Posterior distributions of parameters for the best supported scenario (scenario 6) of the demographic history of *Quercus fabri* with approximate Bayesian computation. Relative median absolute errors (RMAE) based on 500 pseudo-observed data are also indicated for each parameter.

| Parameter | Mean | Median | Mode | 5% lower | 95% high | RMAE |
| --- | --- | --- | --- | --- | --- | --- |
| N1 | 7.60×10^4^ | 7.83×10^4^ | 8.23×10^4^ | 4.68×10^4^ | 9.65×10^4^ | 0.150 |
| N2 | 6.08×10^4^ | 6.19×10^4^ | 6.87×10^4^ | 2.33×10^4^ | 9.27×10^4^ | 0.194 |
| N3 | 8.62×10^4^ | 8.85×10^4^ | 9.03×10^4^ | 6.48×10^4^ | 9.80×10^4^ | 0.145 |
| t1(generations) | 9.36×10^2^ | 6.72×10^2^ | 3.36×10^2^ | 1.02×10^2^ | 2.85×10^3^ | 0.161 |
| r | 4.49×10^-1^ | 4.45×10^-1^ | 4.55×10^-1^ | 2.16×10^-1^ | 7.09×10^-1^ | 0.065 |
| ta | 4.98×10^4^ | 4.99×10^4^ | 5.14×10^4^ | 1.53×10^4^ | 8.29×10^4^ | 0.212 |
| NA | 6.97×10^3^ | 3.11×10^3^ | 7.46×10^2^ | 4.97×10^2^ | 2.60×10^4^ | 0.185 |
| µ  p | 3.13×10^-5^  2.94×10^-1^ | 2.87×10^-5^  3.00×10^-1^ | 2.46×10^-5^  3.00×10^-1^ | 1.67×10^-5^  2.69×10^-1^ | 5.44×10^-5^  3.00×10^-1^ | 0.157  0.109 |

NA, represents the ancestral effective population size. N1, N2 and N3, represent the current population size of Group1, Group 2 and Group 3, respectively.

ta and t1: divergence times for the depicted event.

µ: mutation rate (per generation per locus).

**Table S7** Results of Mantel tests between genetic (GeD) and geographical (GD) distance, and genetic (GeD) and ecological (ED) distances for *Quercus fabri* and three groups based on nuclear microsatellite data.

|  | Matrix | *R* | *p* |
| --- | --- | --- | --- |
| species | GeD–GD | 0.4445 | **0.001** |
|  | GeD–ED | 0.6359 | **0.001** |
|  | GeD–GD (ED partial) | 0.0662 | 0.378 |
|  | GeD–ED (GD partial) | 0.5109 | **0.003** |
| Group 1 | GeD–GD | 0.6605 | 0.146 |
|  | GeD–ED | 0.2421 | 0.218 |
|  | GeD–GD (ED partial) | 0.6670 | 0.119 |
|  | GeD–ED (GD partial) | -0.2703 | 0.445 |
| Group 2 | GeD–GD | -0.0875 | 0.786 |
|  | GeD–ED | -0.1811 | 0.662 |
|  | GeD–GD (ED partial) | 0.0338 | 0.914 |
|  | GeD–ED (GD partial) | -0.1626 | 0.616 |
| Group 3 | GeD–GD | 0.4007 | **0.002** |
|  | GeD–ED | -0.1053 | 0.564 |
|  | GeD–GD (ED partial) | 0.4148 | **0.002** |
|  | GeD–ED (ED partial) | -0.1570 | 0.385 |

**Table S8** Partitioning of the genetic variation in *Quercus fabri* purely associated with climate and geography using redundancy analysis (RDA) and partial RDA. The proportion of genetic variation that could not be partitioned due to the collinearity between geographic and climatic variables (climate + geography) is also shown.

| Genetic variation | Partitioned variance | Proportion constrained (%) | *P*-value |
| --- | --- | --- | --- |
| Total variance | 0.2184 |  |  |
| Full model | 0.1243 | 56.91 | 0.001*** |
| Pure climate | 0.0474 | 21.71 | 0.007** |
| Pure geography | 0.0499 | 22.84 | 0.002** |
| Climate + geography | 0.0270 | 12.36 |  |

**Table S9** Descriptive statistics based on principle components analysis (PCA) of the selected variables. The variables that mainly explained each axis are shown in bold.

| Environmental variable | PC1 | PC2 | PC3 |
| --- | --- | --- | --- |
| BIO1 | 0.877 | 0.386 | -0.277 |
| BIO4 | 0.678 | -0.611 | -0.360 |
| BIO11 | 0.743 | **0.644** | -0.179 |
| BIO12 | 0.791 | 0.075 | **0.579** |
| BIO15  BIO17 | **-0.911**  0.901 | 0.270  -0.241 | 0.099  0.280 |
| Eigenvalue | 4.050 | 1.073 | 0.662 |
| Explained variance (%) | 67.50 | 17.88 | 11.03 |
