# Supplementary material for: Spatial Genetic Structure and Demographic History of the Dominant Forest Oak Quercus fabri Hance in Subtropical China
Source: Front Plant Sci. 2021 Feb 4;11:583284. doi: 10.3389/fpls.2020.583284 (PMC7889815; doi:10.3389/fpls.2020.583284)
Supplement: Supplementary Note 1 — Microsatellite genotype dataset used in this study. [file Data_Sheet_1.PDF]

DIYabc analysis: prior distributions of the parameters with different settings.

Parameter	Minimum	Maximum
Effective population size		
N1	1000	1000000/10000000
N2	1000	1000000/10000000
N3	1000	1000000/10000000
NA	100	1000000/10000000
N4	1000	1000000/10000000
Time scale in generations		
ta	5000	100000
t1	10	5000
Mutation model		
Mean mutation rate	1.00E-06	1.00E-04
Individual locus mutation rate	1.00E-07	1.00E-03
Mean coefficient P	1.00E-01	3.00E-01
Individual locus coefficient P	1.00E-02	9.00E-01
Mean SNI rate	0	0
Individual locus SNI rate	0	0

Principal component analysis results for different parameters settings (above mentioned) obtained along the first three axes for the pre-evaluate scenarios and prior distributions in DIYabc.

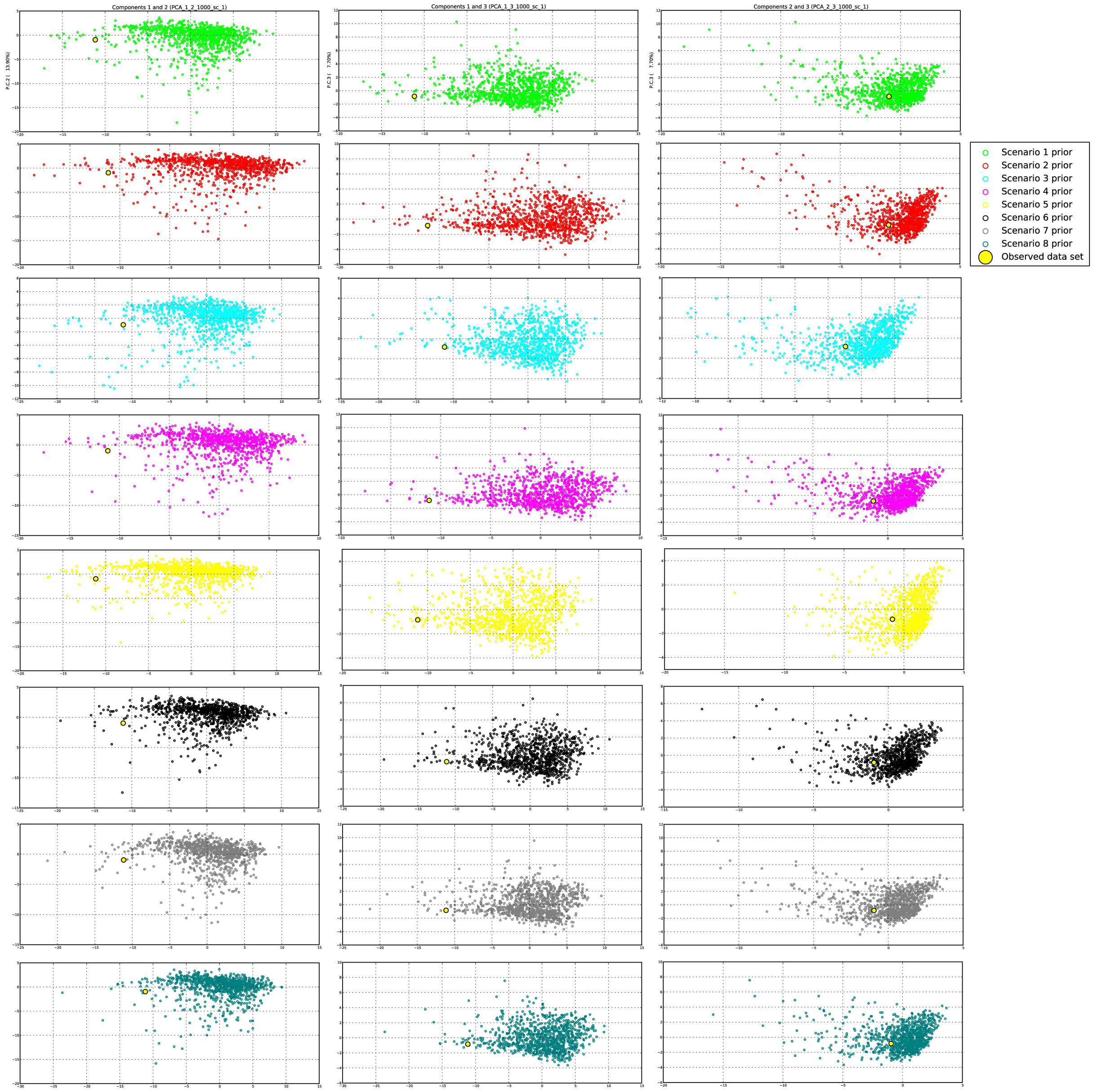

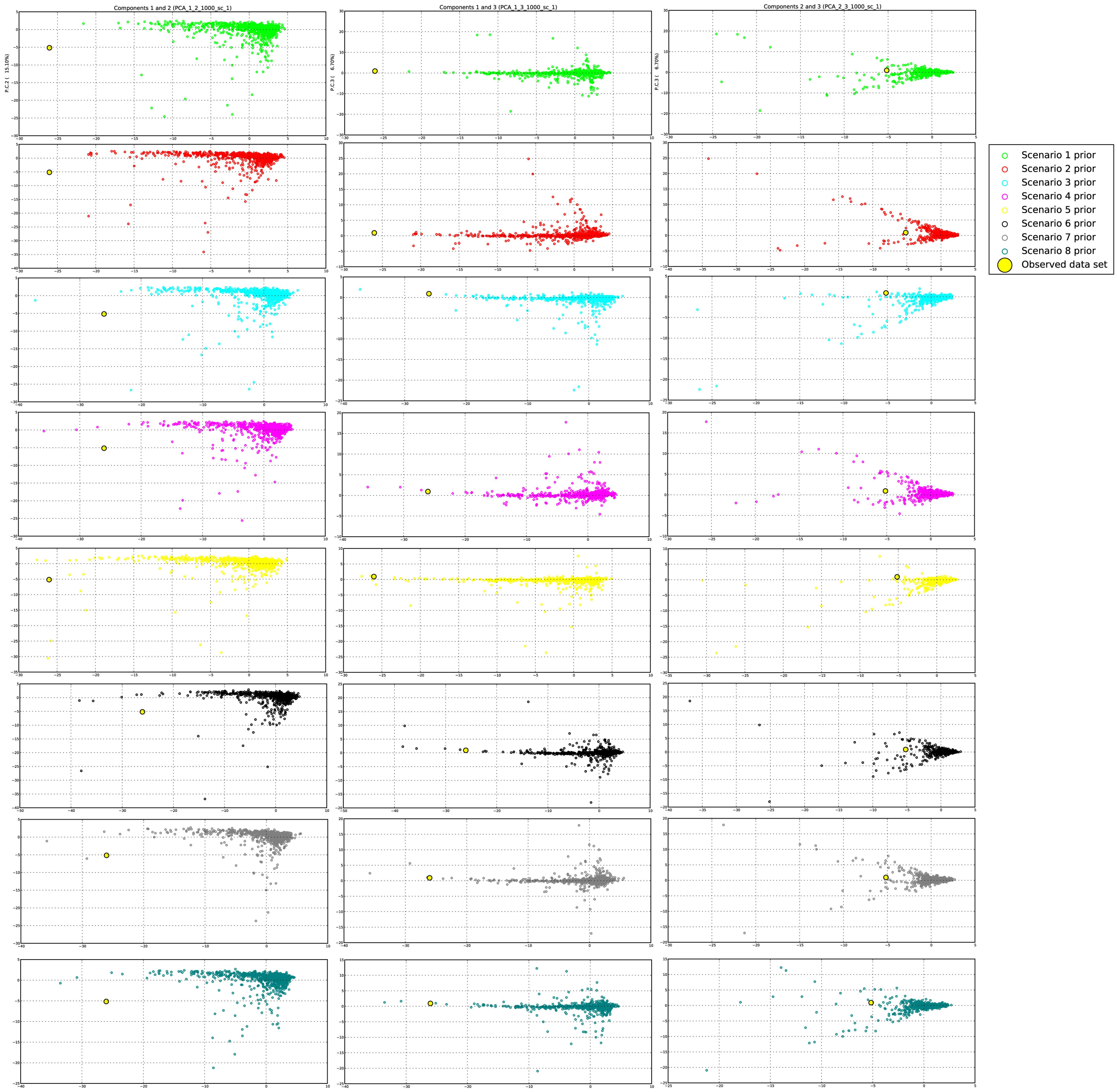

Components 1 and 2 (PCA_1_2_1000_sc_1)
Components 1 and 3 (PCA_1_3_1000_sc_1)
Components 2 and 3 (PCA_2_3_1000_sc_1)
P.C.2 (15.10%)
P.C.3 (6.70%)
Scenario 1 prior
Scenario 2 prior
Scenario 3 prior
Scenario 4 prior
Scenario 5 prior
Scenario 6 prior
Scenario 7 prior
Scenario 8 prior
Observed data set
